# Coupling proteostasis and *de novo* purine biosynthesis of PSMD14 fuels glioblastoma progression and chemoresistance

**DOI:** 10.7150/thno.124409

**Published:** 2026-01-01

**Authors:** Jiazheng Wang, Qun Cao, Zhikai Li, Xuxiu Lu, Zhuo Li, Chenghui Guo, Yuan Pan, Qing Zhang, Wenjie Li, Guo Xiang, Anjing Chen

**Affiliations:** 1 Department of Neurosurgery, Qilu Hospital, Cheeloo College of Medicine and Institute of Brain and Brain-Inspired Science, Shandong University, Jinan 250012, Shandong, China.; 2 Jinan Microecological Biomedicine Shandong Laboratory and Shandong Key Laboratory of Brain Health and Function Remodeling, Jinan 250012, Shandong, China.; 3 Department of Radiation Oncology, Cheeloo College of Medicine, Shandong University, Jinan 250012, Shandong, China.; 4 Rizhao People's Hospital, Rizhao 276800, Shandong Province, China.; 5 Medical Integration and Practice Center, Cheeloo College of Medicine, Shandong University, Jinan, China.

**Keywords:** glioblastoma, PSMD14, deubiquitination, IMPDH2, *de novo* purine biosynthesis, thiolutin

## Abstract

**Background:** Glioblastoma multiforme (GBM) is a highly aggressive primary brain tumor characterized by rapid proliferation, profound invasiveness, and resistance to conventional therapies. Deubiquitinating enzymes (DUBs), crucial regulators of protein homeostasis, have recently been implicated in GBM pathogenesis. However, the specific DUBs that play central roles in GBM pathogenesis and their exact molecular mechanisms remain to be further elucidated.

**Methods:** We systematically analyzed GBM datasets and clinical samples to identify differentially expressed DUBs. Functional experiments, including genetic manipulation, immunoprecipitation coupled mass spectrometry (IP-MS), comprehensive metabolic assays, mitochondrial function assessments, and orthotopic mouse models, were conducted.

**Results:** Here, we identified PSMD14 as a protein significantly upregulated in GBM, with a close correlation to poor prognosis of patients. Mechanistic exploration revealed that PSMD14 stabilized IMPDH2, the rate-limiting enzyme of purine nucleotide biosynthesis, by selectively removing K48-linked polyubiquitin chains. When PSMD14 is inhibited genetically or pharmacologically, IMPDH2 stability diminishes, causing impaired nucleotide metabolism, mitochondrial dysfunction, increased DNA damage signaling, and reduced tumor malignancy. Importantly, these metabolic issues can be reversed by exogenous guanosine, highlighting the key role PSMD14 in metabolic regulation. In translational medicine, the PSMD14 inhibitor, Thiolutin, curbed GBM progression *in vitro* and *in vivo* by disrupting the *de novo* purine biosynthesis and resulting in mitochondrial fragmentation. Moreover, Thiolutin synergized with TMZ to overcome resistance and boost efficacy. This study reveals a new GBM metabolic axis and presents a promising PSMD14-targeting therapy.

**Conclusions:** PSMD14-IMPDH2 axis serves as a crucial hub integrating post-translational modifications and metabolic homeostasis in GBM. Targeting PSMD14 enhances therapeutic sensitivity, presenting a promising strategy to overcome TMZ resistance and improve GBM treatment efficacy.

## Introduction

Glioblastoma multiforme (GBM) is the most aggressive brain tumor in adults, posing a significant clinical challenge due to rapid growth, invasiveness, and resistance to treatments like surgery, radiation, and temozolomide (TMZ) chemotherapy 1, 2, resulting in a median survival of less than 15 months 3. This highlights the urgent need to understand GBM's molecular mechanisms and resistance. A key characteristic of GBM is the disruption of cellular homeostasis 4, particularly proteostasis, which involves the ubiquitin-proteasome system (UPS), crucial for protein stability and cellular function 5, 6. Despite the UPS's critical role in GBM 7, the identification and regulatory mechanisms of deubiquitinating enzymes within the UPS remain poorly understood.

Our previous studies have highlighted the PSMD family, integral components of the 26S proteasome, as significant players in GBM malignancy 8. PSMD14, uniquely functions as the proteasome's intrinsic DUB, selectively removing ubiquitin chains from specific substrates, thus enhancing their stability and cellular function. PSMD14 has been implicated in stabilizing key oncogenic proteins, including β-catenin 9, to promote malignant phenotypes. Notably, PSMD14 is significantly overexpressed in GBM, yet the key regulatory pathways governing its function remain to be elucidated 8, 10.

Rapidly proliferating cancer cells, like those in GBM, impose substantial metabolic demands, particularly on nucleotide metabolism, to sustain cellular proliferation and maintain genomic stability. IMPDH2, a critical rate-limiting enzyme in the purine *de novo* synthesis pathway, is essential for producing guanine triphosphate (GTP), vital for DNA/RNA synthesis, ribosomal biogenesis, and nucleolar function 11. Elevated IMPDH2 expression in GBM ensures sufficient GTP supply and maintains mitochondrial bioenergetics and cellular metabolic stability 12. Disruption of IMPDH2 expression profoundly affects mitochondrial integrity and energy production 13, 14, triggering nucleolar stress, DNA damage responses, and inhibition of tumor proliferation.

In this study, we uncover a pivotal regulatory axis governed by PSMD14 that stabilizes IMPDH2 through the targeted removal of K48-linked ubiquitin chains. This novel mechanism not only integrates post-translational modifications with metabolic homeostasis but also offers a fresh viewpoint on cancer metabolism. Our findings elucidate how PSMD14's deubiquitination of IMPDH2 bolsters nucleotide synthesis and mitochondrial function, creating a metabolic link that fuels GBM's proliferation and invasiveness. Importantly, we reveal the therapeutic potential of pharmacologically inhibiting PSMD14 with Thiolutin 15, which significantly disrupts tumor metabolism and enhances TMZ's efficacy 16, 17. Our research highlights PSMD14's crucial role in *de novo* amino acid synthesis and energy metabolism, positioning Thiolutin as a promising therapeutic agent to enhance treatment efficacy and overcome TMZ resistance in GBM 18.

## Materials and Methods

### Data availability

The datasets and materials generated or analyzed in this study are available from the corresponding author upon reasonable request. All relevant data have been included in the article and supplementary files. Any additional information can be obtained from the corresponding author.

### Ethics approval and consent to participate

The research protocol was reviewed and approved by the Ethical Committee on Scientific Research of Qilu Hospital of Shandong University (approval number: KYLL-2023(ZM)-412), and written informed consent was obtained from each patient included in the study. The patient data were acquired from publicly available datasets, which contained complete informed consent in-formation for the patients. All animal experiments were approved by the Ethics Committee on Animal Experiment of Qilu Hospital of Shandong University (Jinan, China; approval number: DWLL-2023-114).

### Database data and bioinformatics analysis

Bulk RNA-seq data from TCGA and GTEx were downloaded via the GlioVis portal (http://gliovis.bioinfo.cnio.es/). Protein-protein interaction data for the DUB candidates were retrieved from the BioGRID database (https://thebiogrid.org/) to refine the candidate list prior to downstream analyses. Differential expression of DUB genes between GBM and normal brain was computed using edgeR (|log₂FC| > 1, FDR < 0.05) after TMM normalization. Kaplan-Meier survival curves and multivariate Cox regression analyses were performed with the "survival" R package. The single-cell GBM expression dataset GSE84465 was processed with Seurat v4, and Gene Ontology and KEGG pathway enrichment analyses of PSMD14-correlated gene signatures were carried out using Metascape.

### Cell culture

The GBM cell lines LN229, A172 and U118MG were maintained in Dulbecco's Modified Eagle Medium (DMEM; Thermo Fisher Scientific, Waltham, MA, USA) supplemented with 10% fetal bovine serum (FBS; Thermo Fisher Scientific) and 1% penicillin-streptomycin (Thermo Fisher Scientific). Cells were cultured at 37 °C in a 5% CO_2_ incubator. Patient-derived stem-like GBM cells (GBM#P3 and GBM#BG5) were propagated in Neurobasal medium (Gibco/Thermo Fisher Scientific) containing B27 supplement (2%; Thermo Fisher Scientific), EGF (20 ng/mL) and bFGF (10 ng/mL) (PeproTech, East Windsor, NJ, USA). All cell lines were routinely authenticated by short tandem repeat (STR) profiling and confirmed to be mycoplasma-free.

### Transient transfection, lentivirus construction and lentiviral infection

Small interfering RNA (siRNA; GenePharma, Shanghai, China) and plasmid transfections were performed using Lipofectamine 3000 (Thermo Fisher Scientific) according to the manufacturer's protocol, and cells were incubated for 48 h post-transfection. For lentivirus generation, HEK293T cells were co-transfected with the lentiviral transfer vector and packaging plasmids psPAX2 and pCMV-VSV-G. After 48 h, viral supernatants were harvested and used to infect target cells. Infected cells were selected in medium containing puromycin (2 µg/mL; Thermo Fisher Scientific) or blasticidin S hydrochloride (10 µg/mL; Solarbio Life Sciences, Beijing, China) to establish stable cell lines. The siRNA sequences were as follows: siPSMD14-1, 5′-CAAGCCATCTATCCAGGCATT-3′; siPSMD14-2, 5′-CAGATTGATCAATGCTAATAT-3′; siIMPDH2-1,5′-GCCGCUUGGUGGCAUCAUTT-3′; siIMPDH2-2,5′-GGACAGACCUGAGAAGAATT-3′; and a non-targeting control (siNC), 5′-UUCUCCGAACGUGUCACGUTT-3′.

### Cell viability and growth curves

LN229, A172, or GBM#P3 cells were seeded into 96-well plates (3-5×10^3^ cells per well). Cell viability was assessed at 0, 24, 48, 72, and 96 h using a CCK-8 assay kit (Beyotime Biotechnology, Shanghai, China) according to the manufacturer's instructions. Then, 10 µL of CCK-8 reagent was added to each well, and the absorbance at 450 nm was measured after 45 min of incubation at 37 °C. Background absorbance from cell-free control wells was subtracted.

### Cell cycle distribution and programmed cell death

48 hours after siRNA transfection, cells were collected by trypsinisation, washed twice in ice-cold PBS, and fixed overnight in 70% ethanol at -20 °C. After RNase A treatment (100 µg/mL for 30 min at 37 °C), cellular DNA content was stained with propidium iodide (PI, 50 µg/mL) and analysed by flow cytometry (FlowJo v10). Apoptotic fractions were measured using an Annexin V-FITC/PI apoptosis detection kit (BD Biosciences) according to the manufacturer's instructions.

### Migration and invasion assays

Cell motility was examined using 24-well Transwell inserts with 8 µm pores (Corning). For migration assays, 5 × 10^4^ serum-starved cells were seeded in the upper chamber containing DMEM with 1% FBS. For invasion assays, Transwell inserts were pre-coated with 50 µL of growth factor-reduced Matrigel (1 mg/mL), and the assay duration was extended to 48 h. After incubation, non-migrated cells on the upper surface of the membrane were gently removed with cotton swabs; cells that had traversed to the lower side were fixed in methanol, stained with 0.1% crystal violet, and photographed under a 200× objective. Three-dimensional invasion was assessed by embedding pre-formed tumor spheroids (diameter ~300 µm) into Cultrex™ Spheroid Invasion Matrix (Trevigen; Gaithersburg, MD, USA). Radial outgrowth was monitored every 36 h, and invasion distance was calculated relative to the initial spheroid diameter.

### Immunohistochemistry (IHC)

Paraffin-embedded tumor sections (5 µm thick) were deparaffinized, rehydrated, and subjected to heat-induced antigen retrieval in citrate buffer (pH 6.0) for 15 min. Endogenous peroxidase activity was quenched with 3% hydrogen peroxide for 10 min. Sections were blocked with 5% normal goat serum and incubated overnight at 4 °C with primary antibodies against PSMD14 (ab109123; 1:500; Abcam), IMPDH2 (ab129165; 1:500; Abcam) or Ki67 (9449S; 1:1,000; Cell Signaling Technology). After washing, HRP-conjugated secondary antibodies were applied for 30 min, and immunoreactivity was visualized using a DAB chromogen. Nuclei were counterstained with haematoxylin, and sections were dehydrated and mounted.

### Immunofluorescence (IF)

Formalin-fixed, paraffin-embedded (FFPE) GBM tissue sections were deparaffinized, rehydrated, and subjected to antigen retrieval in 10 mM sodium citrate buffer (pH 6.0) at 95 °C for 20 min, followed by cooling in an ice-water bath to room temperature, and non-specific binding was blocked with 5% normal goat serum for 30 min. Slides were incubated overnight at 4 °C with primary antibodies against PSMD14 (ab109123; 1:200; Abcam), IMPDH2 (ab129165; 1:200; Abcam) and Ki67 (9449S; 1:400; Cell Signaling Technology). After equilibration to room temperature, primary antibody was detected with Goat Anti-Mouse IgG H&L (Alexa Fluor® 488) (ab105177; 1:200; Abcam) and Goat Anti-Rabbit IgG H&L (Alexa Fluor® 594) (ab150088; 1:200; Abcam) was applied for 60 min. Sections were then counterstained with DAPI (0.5 µg/mL, 5 min), mounted in antifade medium, and scanned on a Zeiss Axio Scan.Z1 slide scanner equipped with appropriate fluorescence filter sets.

### Western blotting

Whole-cell extracts were prepared in ice-cold RIPA lysis buffer (Beyotime Biotechnology, Shanghai, China) supplemented with protease and phosphatase inhibitor cocktails (Beyotime Biotechnology, Shanghai, China). Protein concentrations were determined using the Pierce™ BCA Protein Assay (Thermo Fisher Scientific). Equal aliquots of denatured protein lysates (40 µg per lane) were resolved on 10% SDS-PAGE gels and transferred onto Immobilon-P polyvinylidene difluoride (PVDF) membranes (Millipore). Membranes were blocked in 5% (w/v) non-fat milk in TBST for 1 h, then incubated overnight at 4 °C with primary antibodies targeting PSMD14 (4197S; 1:1,000; Cell Signaling Technology), IMPDH2 (36281S; 1:1,000; Cell Signaling Technology), β-Actin (4970S; 1:1,000; Cell Signaling Technology), ubiquitin (20326S; 1:1,000; Cell Signaling Technology), K48-linkage-specific polyubiquitin (8081S; 1:1,000; Cell Signaling Technology), K63-linkage-specific polyubiquitin (5621S; 1:1,000; Cell Signaling Technology), His-Tag (12698T; 1:1000; Cell Signaling Technology), HA-Tag (3724S; 1:1000; Cell Signaling Technology) and Myc-Tag (2276S; 1:1,000; Cell Signaling Technology). After washing, HRP-conjugated secondary antibodies (1:5,000; Thermo Fisher Scientific) were applied for 1 h at room temperature, and bands were visualized using SuperSignal™ West Pico PLUS chemiluminescent substrate on a ChemiDoc MP imaging system (Bio-Rad). Where specified, cells were pre-treated with cycloheximide (25 µg/mL for 9 h; HY-123320; MedChemExpress), MG132 (10 µM for 8 h; #474790; Sigma-Aldrich), or thiolutin (2 µM for 8 h; HY-N6712; MedChemExpress) prior to lysis.

### Silver stain assay and mass spectrometry (MS)

Whole-cell lysates (40 µg each) from PSMD14 immunoprecipitates and corresponding IgG controls were resolved on 10% SDS-PAGE gels, visualized using a rapid silver staining kit (Beyotime Biotechnology, Shanghai, China), and proteins were detected by MS.

### Co-immunoprecipitation (Co-IP)

Cells were lysed in ice-cold NP-40 buffer (50 mM Tris-HCl, pH 7.5, 150 mM NaCl, 1% NP-40, plus protease inhibitors). Cleared lysates (~1 mg protein) were incubated with 2 µg of the appropriate antibody along with Protein G agarose beads (Thermo Fisher Scientific) for 4 h at 4 °C. After extensive washing, bound proteins were eluted in 2× Laemmli sample buffer (Thermo Fisher Scientific), resolved by SDS-PAGE, and analyzed by immunoblotting. For ubiquitination assays, cells were pre-treated with MG132 (10 µM for 8 h; #474790; Sigma-Aldrich) prior to lysis.

### Measurement of xanthine/hypoxanthine levels, ATP levels and seahorse XF analysis

Xanthine / hypoxanthine levels and ATP levels were determined as described in the manual provided by the Amplex Red Xanthine/Xanthine Oxidase Assay Kit (Beyotime Biotechnology, Shanghai, China) and ATP Assay Kit (Beyotime Biotechnology, Shanghai, China). Real-time cellular respiration and glycolysis were measured using a Seahorse XF24 Analyzer (Agilent Technologies). LN229 and GBM#P3 cells were seeded in XF24 cell culture microplates at densities of 2.5 × 10^4^ and 3 × 10^4^ cells per well, respectively, 24 h before the assay to reach ~80% confluency. One hour prior to the assay, the culture medium was replaced with Seahorse XF Base Medium (Agilent Technologies) adjusted to pH 7.4 and supplemented with 25 mM D-glucose, 2 mM L-glutamine, and 1 mM sodium pyruvate (200 µL per well). Plates were incubated at 37 °C in a non-CO₂ incubator for 1 h to allow temperature and pH equilibration, and the sensor cartridge was concurrently hydrated and calibrated according to the manufacturer's instructions. For the Mito Stress Test, oligomycin (1 µM; ATP synthase inhibitor), FCCP (1.5 µM; uncoupler), and a rotenone/antimycin A mixture (0.5 µM each; Complex I and III inhibitors) were sequentially injected into ports A, B, and C, respectively. Oxygen consumption rate (OCR) and extracellular acidification rate (ECAR) were recorded every 5 min (mix for 3 min, wait for 2 min, measure for 3 min) for three cycles at basal conditions and after each injection. At the end of the assay, cells were lysed in the culture plate with RIPA buffer (Beyotime), and total protein was quantified using a BCA assay. OCR and ECAR values were normalized to total protein content per well.

### JC-1 mitochondrial membrane potential assay

Mitochondrial membrane potential (ΔΨm) was evaluated using the cationic dye JC-1 (Beyotime) via both fluorescence microscopy and flow cytometry. LN229 and GBM#P3 cells were seeded on poly-L-lysine-coated glass coverslips (1 × 10^5^ cells per coverslip) and subjected to the indicated treatments for 24-48 h. Cells were then incubated with JC-1 working solution (5 µM in serum-free DMEM) for 20 min at 37 °C in the dark. After two washes with warm JC-1 buffer, coverslips were mounted in Live Cell Imaging Solution and immediately visualized under a Leica DMi8 inverted fluorescence microscope equipped with FITC (green, monomer) and TRITC (red, aggregate) filter sets. The red-to-green fluorescence ratio was quantified using ImageJ software.

For quantitative flow cytometry analysis, treated cells grown in 6-well plates were harvested with 0.05% trypsin-EDTA, washed twice in ice-cold PBS, and stained with JC-1 in suspension as described above. After washing, cells were resuspended in 500 µL of JC-1 buffer and analyzed within 15 min by flow cytometry (FL1 channel at 530 nm for green monomers; FL2 channel at 585 nm for red aggregates). A minimum of 10,000 events per sample was collected. Data were processed using FlowJo v10.

### EdU incorporation assay

Nascent DNA synthesis was measured using a Click-iT™ EdU Cell Proliferation Kit (Yeasen). Cells in logarithmic growth phase were seeded onto glass coverslips at 1 × 10^5^ cells per well in 24-well plates and treated as indicated. A 10 µM EdU pulse was administered for 2 h, after which cells were fixed in 4% paraformaldehyde for 15 min at room temperature and permeabilized with 0.5% Triton X-100 for 10 min. Click chemistry was performed in the dark for 30 min according to the manufacturer's protocol to conjugate Alexa Fluor™ 594 azide to the incorporated EdU. Nuclei were counterstained with Hoechst 33342 (5 µg/mL, 5 min). Coverslips were washed with PBS containing 3% BSA between steps, mounted with antifade medium, and imaged on a Leica DMi8 fluorescence microscope.

### Immunocytochemistry (ICC)

Following experimental treatments, cells grown on coverslips were fixed in 4% paraformaldehyde for 15 min and permeabilized with 0.3% Triton X-100 for 10 min. Non-specific binding was blocked by incubating samples in 5% bovine serum albumin (BSA) in PBS for 30 min. Cells were then incubated overnight at 4 °C with primary antibodies diluted in blocking buffer. For example, a typical staining protocol included anti-γH2AX (Cat# 7631S; 1:200; Cell Signaling Technology), anti-Nucleostemin (ab70346; 1:200; Abcam), and the mitochondrial dye MitoTracker (Cat# 9074S; 500 nM; Cell Signaling Technology). After washing, Alexa Fluor® 488- and/or 594-conjugated secondary antibodies (ab150077 and ab150080; 1:200; Abcam) were applied for 1 h at room temperature in the dark. Nuclei were counterstained with DAPI (1 µg/mL, 5 min; Beyotime). Finally, slides were mounted with ProLong™ Gold Antifade Mountant and examined under an Olympus IX83 inverted fluorescence microscope equipped with a CCD camera.

### Xenografts and drug treatment

Luciferase-expressing GBM#P3 cells (3 × 10^5^ cells in 10 µL PBS) were stereotactically injected into the right striatum of 4-week-old male BALB/c-nu/nu mice (Shanghai SLAC Laboratory Animal Co., Ltd., Shanghai, China) at coordinates 1 mm anterior, 2 mm lateral to bregma, and 2.5 mm depth. Tumor burden was monitored weekly using an IVIS Spectrum imaging system after intraperitoneal injection of D-luciferin (150 mg/kg; Yeasen). Seven days after implantation, mice were randomized into groups and treated with thiolutin (2 mg/kg, i.v., every other day; HY-N6712; MedChemExpress), temozolomide (50 mg/kg, i.g., on days 1-5 of each 7-day cycle; HY-17364; MedChemExpress), or a combination of both agents. Body weight and neurological status were recorded throughout the treatment period. Animals were sacrificed upon reaching humane endpoints, and brains were fixed in 4% paraformaldehyde for histological evaluation.

## Results

### PSMD14 is upregulated in GBM and correlates with increased tumor invasiveness

To systematically identify deubiquitinating enzymes (DUBs) that potentially drive GBM progression, we performed integrative transcriptomic analyses combining differential expression profiling of GBM versus normal brain tissues with a curated list of DUB genes. Differential expression analysis identified significantly upregulated and downregulated genes in GBM compared with normal brain tissue (Figure S1A). Intersection of these differentially expressed genes (DEGs) with a curated list of DUBs yielded 2,869 candidate genes potentially relevant to GBM pathogenesis (Figure S1B).

PSMD14, a member of the JAB1/MPN/Mov34 (JAMM) metalloprotease family and the only intrinsic DUBs embedded in the 19S regulatory particle of the 26S proteasome, emerged as the top candidate with significantly elevated expression in GBM (Figure S1C-E) 19. Analysis of The Cancer Genome Atlas (TCGA) glioma cohorts revealed a stepwise increase in PSMD14 mRNA levels with ascending tumor grade (Figure S1F), with the highest expression observed in WHO grade IV GBM (Figure S1G). Clinically, elevated PSMD14 expression was associated with key oncogenic alterations frequently observed in GBM, including EGFR amplification, PTEN deletion, and TP53 mutation (Figure S1H, I) 20. Moreover, high PSMD14 expression correlated with significantly shortened overall survival (Figure S1J), and receiver operating characteristic (ROC) curve analysis demonstrated its potential as a prognostic biomarker (Figure S1K).

**Figure 1:**
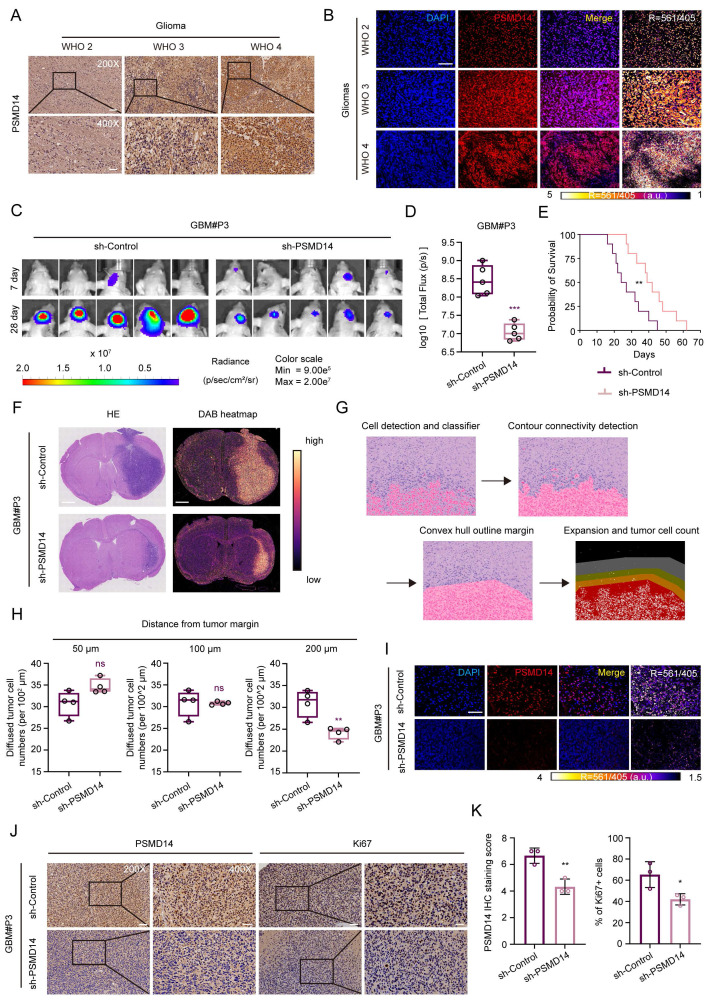
** Elevated PSMD14 expression underpins glioblastoma progression by promoting tumor growth and dispersal *in vivo*.** (A) Representative immunohistochemical (IHC) images for PSMD14 in human GBM tissues (n = 4 per group). Scale bar = 100 μm. (B) Representative immunofluorescence (IF) images of PSMD14 in GBM tissues (n = 4 per group). Scale bar = 100 μm. (C) Tumor formation in an orthotopic GBM xenograft model after PSMD14 knockdown. Shown are representative images of tumor-bearing mice at endpoint (n = 5 per group). (D) Quantification of tumor burden in xenograft-bearing mice based on total fluorescence intensity per mouse. Data are presented as mean ± SD (n = 5 per group). ***P <0.001 (Independent-sample Student-T test). (E) Kaplan-Meier survival curves of mice with intracranial GBM xenografts from PSMD14-knockdown or control cells (n = 10 per group). Statistical significance was assessed by the log-rank test. (F) Representative hematoxylin and eosin (HE) staining and DAB heatmap for PSMD14 in orthotopic xenograft brain tumors. Scale bar = 2.5 mm. (G) Schematic overview of the integrated data processing pipeline. (H) Quantification of disseminated tumor cells at defined distances from the primary tumor margin in brain sections, determined by cell counting on serial sections (n = 4 per group). Data are presented as mean ± SD. **P <0.01 (Independent-sample Student-T test). (I) Representative IF images of xenograft tumor tissues. Scale bar = 100 μm. (J) Representative IHC images of PSMD14 in brain tumor sections from xenografted mice. Scale bar = 100 μm. (K) Quantification of PSMD14 IHC signal (H-score) in xenograft tumor tissues across multiple fields (n = 3 per group). Data are presented as mean ± SD. *P < 0.05, **P < 0.01 (Independent-sample Student-T test).

To validate these findings at the protein level, we performed immunohistochemical (IHC) and immunofluorescence (IF) analysis of PSMD14 expression in human GBM tissues. Consistent with transcriptomic data, GBM specimens showed significantly elevated PSMD14 protein expression, as quantified by H-score (Figure 1A, B; Figure S1L). Comparative analysis revealed consistently elevated PSMD14 expression across multiple GBM cell lines relative to normal human astrocyte (NHA) (Figure S2A, B). To explore whether PSMD14 expression is spatially enriched in specific tumor compartments, we next analyzed anatomically resolved RNA-seq datasets from GBM surgical specimens. Violin plots demonstrated that PSMD14 mRNA levels were significantly higher in leading edge (LE), infiltrating tumor (IT), and cellular tumor (CT) regions 21, compared with necrotic and microvascular zones (CTpan, CTpnz, CTbv, CTmvp) (Figure S2C) 22. These results suggest that PSMD14 is preferentially expressed in infiltrative and proliferative niches of the tumor, implicating a role in glioma invasion and core growth.

We next further identify the functional consequence of PSMD14 dysregulation in GBM *in vivo*. Knockdown of PSMD14 in patient-derived glioma primary cells GBM#P3 significantly suppressed intracranial tumor growth in orthotopic xenografts, as evidenced by bioluminescence imaging and histological analysis (Figure 1C, D**;** Figure S2D, E). Kaplan-Meier survival analysis demonstrated that PSMD14 knockdown significantly prolonged survival compared to controls (Figure 1E). Considering the spatial transcriptomic findings and the diffuse nature of GBM, we further examined whether PSMD14 depletion affected tumor infiltration. Following established histopathological approaches, we performed hematoxylin and eosin (HE) staining on paraffin-embedded brain sections and delineated tumor borders to quantify the extent of peritumoral cell dispersion 23. Specifically, the number and migration distance of individual tumor cells beyond the defined boundary were systematically measured across representative fields. This analysis revealed that PSMD14 knockdown markedly reduced both the frequency and radial range of infiltrative cells relative to control xenografts (Figure 1F-H**;** Figure S2F, G). Immunofluorescence and IHC analyses revealed markedly reduced PSMD14 protein expression in PSMD14-knockdown tumors compared to controls (Figure 1I-K). Consistent with these findings, immunofluorescence staining showed a significant decrease in Ki67-positive proliferating tumor cells upon PSMD14 depletion (Figure S2F). These data support a role for PSMD14 in facilitating GBM cell dispersal within brain parenchyma, complementing its effect on core tumor growth.

### PSMD14 is required for GBM cell proliferation, invasion, and survival

**Figure 2:**
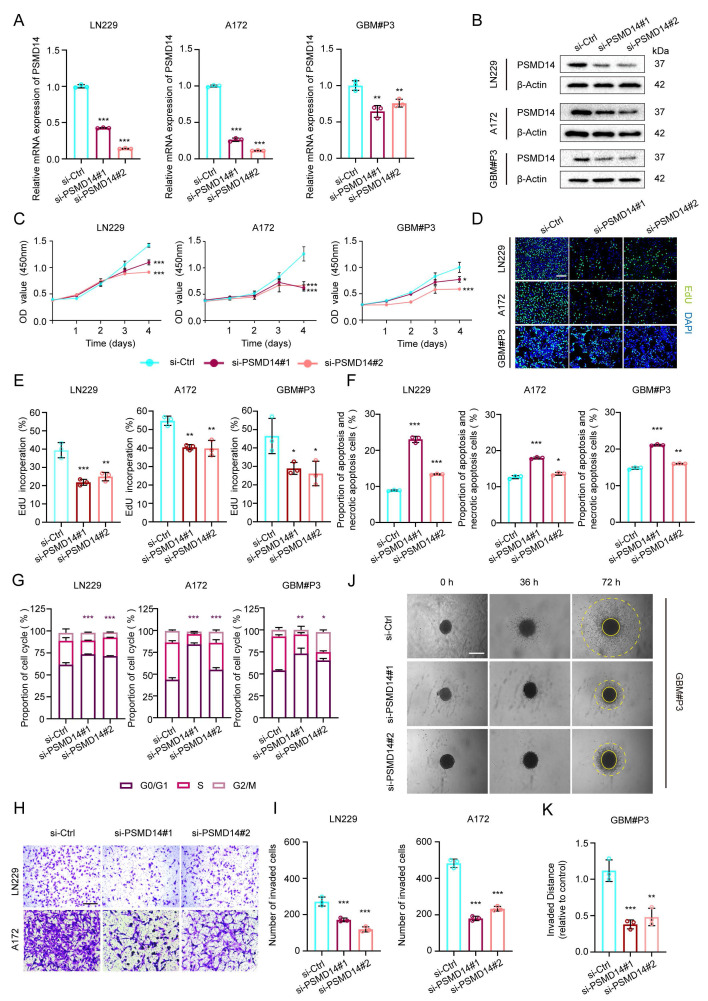
** Knockdown of PSMD14 impairs proliferation, induces apoptosis and cell cycle arrest, and suppresses invasion in GBM cells.** (A) Quantitative real-time PCR (qRT-PCR) analysis of PSMD14 mRNA levels in control versus PSMD14-knockdown GBM cells. Data are presented as mean ± SD (n = 3). **P < 0.01, ***P < 0.001 (one-way ANOVA). (B) Western blot analysis confirming PSMD14 knockdown in GBM cells. (C) Cell viability curves for control and PSMD14-knockdown GBM cells over time. Data are presented as mean ± SD (n = 3). **P < 0.01, ***P <0.001 (two-way ANOVA). (D) Representative images of EdU incorporation assays showing GBM cell proliferation in control and PSMD14-knockdown cells. Scale bar = 100 μm. (E) Quantification of EdU-positive cells in control and PSMD14-knockdown groups. Data are presented as mean ± SD (n = 3). *P < 0.05, **P < 0.01, ***P < 0.001 (one-way ANOVA). (F) Flow cytometric analysis of apoptosis in control and PSMD14-knockdown GBM cells (Annexin V-FITC/PI). The percentage of apoptotic cells is shown as mean ± SD (n = 3). *P < 0.05, **P < 0.01, ***P < 0.001 (one-way ANOVA). (G) Cell cycle distribution of control and PSMD14-knockdown cells. The statistical comparisons and asterisks specifically refer to the G0/G1 fraction among the three groups. Data are presented as mean ± SD (n = 3). *P < 0.05, **P < 0.01, ***P < 0.001 (one-way ANOVA). (H) Representative images of Transwell migration and invasion assays for control and PSMD14-knockdown GBM cells. Scale bar = 100 μm. (I) Quantification of migrated and invaded cells in Transwell assays. Data are presented as mean ± SD (n = 3). ***P < 0.001 (one-way ANOVA). (J) Three-dimensional (3D) spheroid invasion assays demonstrating the invasive capacity of GBM spheroids in Matrigel for control versus PSMD14-knockdown groups. Scale bar = 100 μm. (K) Quantification of relative invasion distance in 3D spheroid assays. Data are presented as mean ± SD (n = 3). **P < 0.01, ***P < 0.001 (one-way ANOVA).

**Figure 3:**
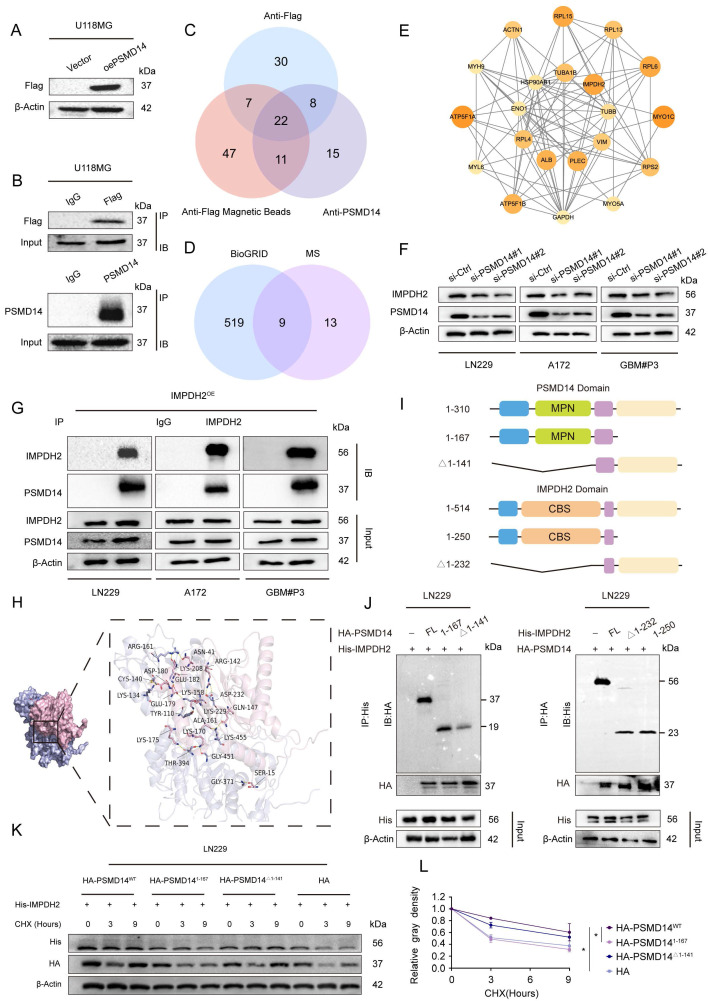
** PSMD14 interacts with IMPDH2 and regulates its protein stability in GBM.** (A) Western blot confirming overexpression of exogenous Flag-tagged PSMD14 in U118MG cells. (B) The Co-IP assay of PSMD14 from U118MG cells. (C) Mass spectrometry identification of proteins co-precipitated with PSMD14 in U118MG cells, followed by intersection analysis to identify common candidate interactors. (D) Bioinformatic overlap of the PSMD14 interactome identified by mass spectrometry with known interactions in the BioGRID database. (E) Network analysis of high-confidence interacting proteins with PSMD14. (F) Western blotting analysis to detect changes in IMPDH2 expression after knockdown of PSMD14. (G) The Co-IP assay confirming the interaction between PSMD14 and IMPDH2 in GBM cells. (H) Structural docking model of PSMD14 and IMPDH2 showing the predicted binding interface rendered with PyMOL (PSMD14 in pink, IMPDH2 in purple). (I) Schematic diagram of the PSMD14 and IMPDH2 domain structures and the design of truncation mutants. (J) The Co-IP analysis using PSMD14 and IMPDH2 truncation mutants, identifying the regions required for their interaction. (K) Cycloheximide (CHX) chase assay in control and PSMD14-knockdown GBM cells, evaluating IMPDH2 protein stability over time. (L) Quantification of IMPDH2 protein half-life from CHX chase assays. Data are presented as mean ± SD (n = 3). *P < 0.05 (one-way ANOVA).

To delineate the functional relevance of PSMD14 in GBM pathobiology, we systematically examined the consequences of PSMD14 depletion across multiple established and primary GBM cell models. siRNA-mediated silencing of PSMD14 in LN229, A172, and primary patient-derived GBM#P3 cells led to a marked reduction in both mRNA and protein levels, confirming efficient knockdown (Figure 2A, B). Functionally, loss of PSMD14 profoundly impaired cell viability and DNA synthesis, as evidenced by significant reductions in CCK-8 and EdU incorporation assays (Figure 2C-E). Flow cytometry analyses revealed that PSMD14 deficiency induced pronounced G0/G1 phase cell cycle arrest, accompanied by increased apoptosis rates (Figure 2F, G**;** Figure S3A, B). Beyond its role in cell proliferation, PSMD14 loss resulted in a dramatic suppression of GBM cell invasive capacity, as demonstrated by both Transwell invasion assays (Figure 2H, I) and 3D spheroid outgrowth (Figure 2J, K). Consistently, the observed defects in proliferation, cell cycle progression, apoptosis, and invasiveness upon PSMD14 silencing underscore its multifaceted regulatory role in driving tumor aggressiveness. Taken together, our results firmly position PSMD14 as an essential regulator orchestrating the key oncogenic phenotypes of GBM.

### PSMD14 interacts with IMPDH2 and maintains its protein stability in GBM cells

To comprehensively elucidate the downstream molecular effectors of PSMD14 in GBM, we employed an unbiased proteomic approach by performing co-immunoprecipitation followed by mass spectrometry (Co-IP/MS) in GBM cells stably overexpressing Flag-tagged PSMD14 (Table S1). This screen identified 22 high-confidence interacting proteins (Figure 3A-C**;** Figure S4A-D), of which nine overlapped with previously reported interactors in the BioGRID database (Figure 3D). Network analysis highlighted IMPDH2 — a rate-limiting enzyme for *de novo* GTP biosynthesis and a known regulator of purine metabolism — as a central node within the PSMD14 interactome 24, implicating its potential role in GBM metabolic adaptation and therapeutic resistance (Figure 3E**;** Figure S4E-H) 25. Furthermore, knockdown of PSMD14 led to a marked reduction in IMPDH2 expression (Figure 3F).

By integrating these proteomic and functional analyses, we rationally selected IMPDH2 for further mechanistic investigation. As following, IMPDH2 was confirmed as a direct PSMD14 interactor by reciprocal co-immunoprecipitation and high-resolution immunofluorescence colocalization (Figure 3G**;** Figure S4I). Computational docking simulations predicted the interaction interface between PSMD14 and IMPDH2 (Figure 3H), which was subsequently validated using domain truncation mutants. These experiments pinpointed the MPN domain of PSMD14 and the CBS domain of IMPDH2 as essential for their physical interaction (Figure 3I, J**;** Figure S4J, K) 26. Furthermore, knockdown of PSMD14 in GBM cell lines abolished PSMD14-mediated stabilization of IMPDH2 protein, indicating the functional relevance of this interaction (Figure 3K, L).

### PSMD14 removes K48-linked polyubiquitin of IMPDH2 via its MPN domain

**Figure 4:**
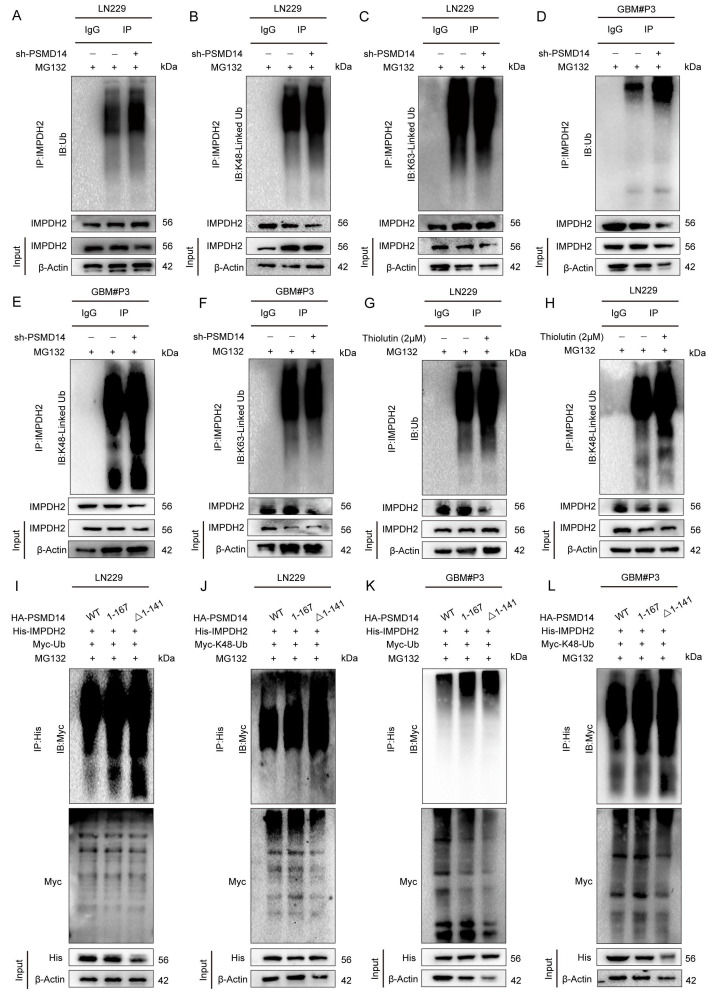
** PSMD14 regulates IMPDH2 stability through selective removal of K48-linked ubiquitin chains.** (A-C) Western blotting analysis of ubiquitin conjugates in LN229 cells with PSMD14 knockdown. (D-F) Western blotting analysis of ubiquitin conjugates in GBM#P3 cells with PSMD14 knockdown. (G, H) Ubiquitination status of IMPDH2 upon PSMD14 inhibition. GBM cells were treated with the PSMD14 inhibitor thiolutin in the presence of MG132. (I-L) Domain mapping of PSMD14's deubiquitinating function. GBM cells were co-transfected with wild-type PSMD14 or N-terminal truncation mutants along with plasmids encoding Myc-tagged ubiquitin.

To dissect the molecular mechanism by which PSMD14 regulates IMPDH2 stability, we systematically evaluated the ubiquitination status and turnover of IMPDH2 upon PSMD14 inhibition. Genetic knockdown of PSMD14 in GBM cell lines markedly increased K48-linked polyubiquitination of IMPDH2, without affecting K63-linked polyubiquitin chains (Figure 4A-C). This selective accumulation of K48-linked polyubiquitin was consistently observed across several GBM cell lines, highlighting the specificity and robustness of this regulatory axis 27. Consistently, pharmacological inhibition of PSMD14 using Thiolutin also specifically elevated K48-linked ubiquitination levels of IMPDH2 in these GBM models (Figure 4D-F). However, proteasomal inhibition with MG132 failed to fully restore IMPDH2 protein abundance following PSMD14 depletion, indicating that PSMD14-mediated deubiquitination occurs upstream and is indispensable for IMPDH2 stabilization (Figure 4G, H). Rescue experiments further confirmed the necessity of an intact PSMD14 MPN domain for this regulation. Only wild-type PSMD14, and not the MPN domain-deletion mutants, effectively reduced K48-linked ubiquitination levels of IMPDH2 and restored its protein stability (Figure 4I-L). Collectively, these findings establish that PSMD14 specifically functions to stabilize IMPDH2 protein by catalyzing removal of K48-linked polyubiquitin chains, thus maintaining purine biosynthesis homeostasis and underscoring PSMD14's potential as a therapeutic target in GBM.

### The PSMD14-IMPDH2 axis is necessary for GBM cell proliferation and invasion

To delineate the functional significance of the PSMD14-IMPDH2 axis in GBM, we first analyzed clinical transcriptomic datasets. IMPDH2 expression was significantly upregulated in glioma tissues relative to normal brain (Figure S5A). Notably, independent knockdown of IMPDH2 recapitulated the effects observed upon PSMD14 depletion, resulting in prominent G0/G1 cell-cycle arrest (Figure S5B-E), and elevated apoptosis levels (Figure S5F-G). To further confirm the functional dependence of PSMD14 effects on IMPDH2, ectopic overexpression of IMPDH2 was conducted in PSMD14-silenced GBM cells. IMPDH2 restoration effectively rescued cell proliferation (Figure S5H, I) and DNA synthesis (Figure S5J, K), and invasive capacity (Figure S5L, M) to near control levels. Collectively, these findings robustly position IMPDH2 as a critical downstream mediator of PSMD14-driven oncogenic phenotypes, highlighting the PSMD14-IMPDH2 axis as a promising therapeutic target in GBM.

### The PSMD14-IMPDH2 axis integrates metabolic homeostasis and mitochondrial integrity in GBM

**Figure 5:**
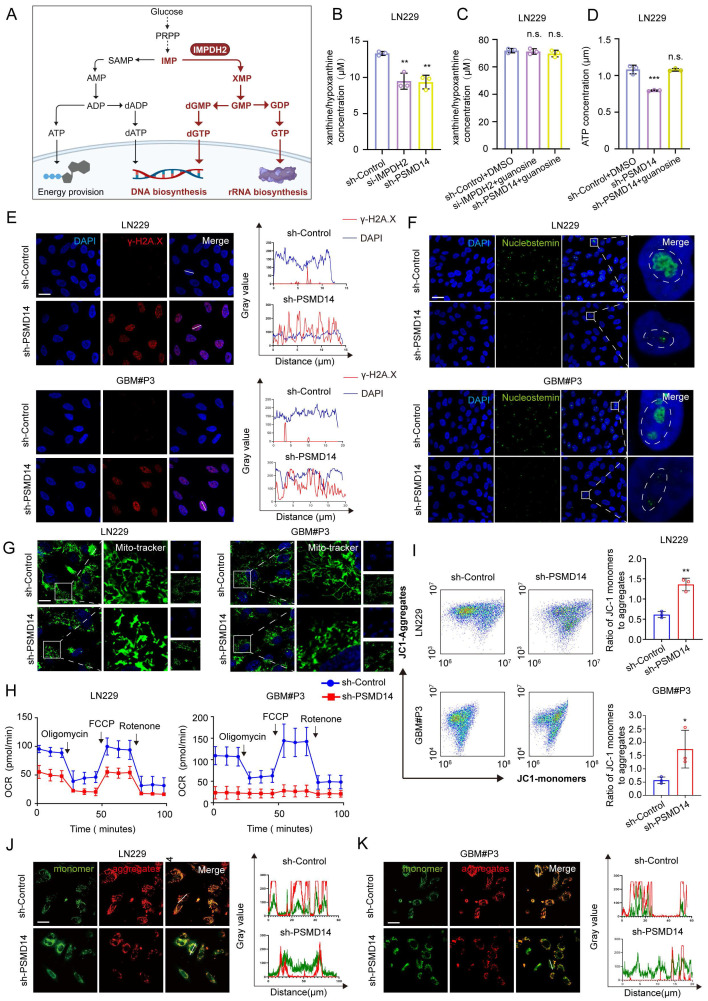
** Loss of PSMD14 disrupts nucleotide metabolism, induces DNA damage and impairs mitochondrial function in GBM cells.** (A) Schematic depiction of metabolic pathways regulated by IMPDH2 in GBM cells. (B-D) Relative levels of metabolites in GBM cells expressing siIMPDH2 or shPSMD14. Data are presented as mean ± SD (n = 3). **P < 0.01, ***P < 0.001 (one-way ANOVA). (E) ICC images showing accumulation of γ-H2AX in GBM cells upon PSMD14 knockdown. Scale bar = 30 μm. (F) ICC images indicating nucleolar stress in PSMD14-knockdown GBM cells. Nucleoli are labeled by nucleostemin. Scale bar = 30 μm. (G) ICC images of mitochondria in PSMD14-knockdown cells. Scale bar = 30 μm. (H) Seahorse extracellular flux analysis of mitochondrial respiration in control and PSMD14-knockdown GBM cells. Data are presented as mean ± SD (n = 3). *P < 0.05, **P < 0.01 (Independent-sample Student-T test). (I) Flow cytometric analysis of mitochondrial membrane potential in control and PSMD14-knockdown cells. Data are presented as mean ± SD (n = 3). *P < 0.05, **P < 0.01 (Independent-sample Student-T test). (J-K) ICC images of JC-1 staining showing changes in mitochondrial membrane potential. Scale bar = 30 μm.

To systematically elucidate the cellular consequences of disrupting the PSMD14-IMPDH2 axis, we conducted a series of metabolic and organellar phenotyping assays across GBM models (Figure 5A). Functional ablation of either PSMD14 or IMPDH2 precipitated a rapid and profound depletion of intracellular purine metabolites, including hypoxanthine and xanthine, demonstrating a collapse of the *de novo* purine biosynthetic pathway (Figure 5B). Notably, supplementation with exogenous guanosine fully rescued the levels of both purine metabolites and cellular ATP (Figure 5C, D), directly linking nucleotide supply to energy homeostasis in GBM cells. Mechanistically, PSMD14-IMPDH2 axis disruption resulted in severe genotoxic and nucleolar stress: γ-H2A. X levels were markedly elevated, indicating widespread DNA damage (Figure 5E**;** Figure S6A), while nucleostemin expression was suppressed, reflecting nucleolar dysfunction (Figure 5F). These findings highlight that impairment of nucleotide biosynthesis not only curtails energy production but also undermines genome maintenance and cellular proliferation. Crucially, our study demonstrates that the impact of PSMD14-IMPDH2 signaling extends well beyond nucleotide pools, fundamentally compromising mitochondrial integrity and bioenergetic capacity. MitoTracker and confocal imaging revealed pronounced mitochondrial network fragmentation and loss of tubular morphology (Figure 5G**;** Figure S6B-D) 28, while Seahorse analysis documented a sharp decline in mitochondrial respiration (Oxygen Consumption Rate, OCR) (Figure 5H; Figure S6E, F) 29. JC-1 staining further confirmed mitochondrial membrane potential depolarization (Figure 5I-K), underscoring a breakdown of the proton gradient and the onset of mitochondrial dysfunction 30.

By systematically interrogating these endpoints, we established that loss of IMPDH2 triggers a multifaceted metabolic crisis: as the gatekeeper of *de novo* GTP biosynthesis, IMPDH2 deficiency precipitates GTP depletion, thereby impairing several GTP-dependent mitochondrial functions—ranging from TCA cycle substrate-level phosphorylation and mitochondrial translation to the maintenance of cristae structure by key GTPases 31. The collapse of both oxidative phosphorylation and glycolysis results in ATP exhaustion, while severe ΔΨm dissipation ultimately tip the balance toward cell death. Collectively, our data reveal that the PSMD14-IMPDH2 axis constitutes a critical regulatory node that synchronizes post-translational modification with nucleotide and energy metabolism, ensuring both genomic integrity and mitochondrial homeostasis. This mechanistic linkage not only underpins the metabolic adaptability of GBM but also identifies the PSMD14-IMPDH2 pathway as a tractable metabolic vulnerability for therapeutic intervention.

### Combined targeting of the PSMD14-IMPDH2 axis potentiates temozolomide efficacy in GBM through dual metabolic and DNA damage vulnerability

**Figure 6:**
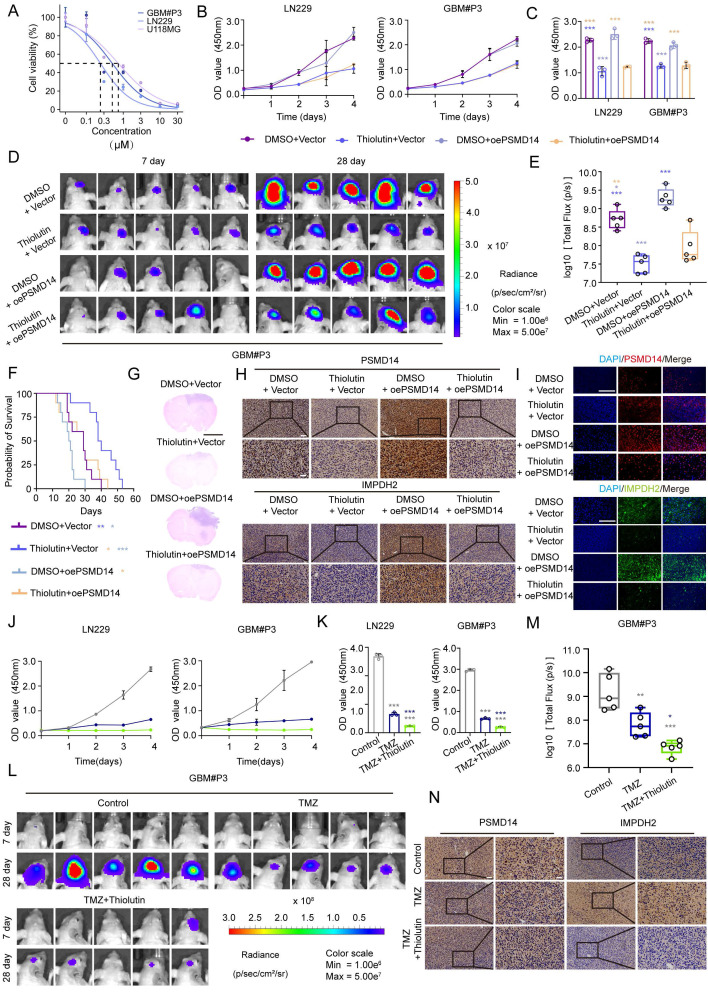
** Pharmacological inhibition of PSMD14 with thiolutin reduces tumor burden and synergizes with temozolomide in GBM.** (A) IC50 curves of Thiolutin in the GBM#P3, U118, and LN229 cell lines. (B) Time-course of cell viability in GBM cells with ectopic PSMD14 overexpression or empty vector control, in the presence of DMSO or thiolutin. (C) Quantification of cell viability (OD values) on Day 4 (n = 3). ***P <0.001 (one-way ANOVA). (D) Representative bioluminescence images of intracranial GBM#P3 xenograft-bearing mice at Days 7 and 28 post-implantation. Mice were treated with vehicle or thiolutin, with or without PSMD14 overexpression in the implanted cells. (E) Quantification of total photon flux from intracranial tumors at Day 28. Data are presented as mean ± SD (n = 5 per group). ***P <0.001 (one-way ANOVA). (F) Kaplan-Meier survival analysis of mice bearing intracranial tumors under the indicated treatments (n = 10 per group). (G) Representative HE is staining of brain sections from tumor-bearing mice across treatment groups. Scale bar = 2.5 mm. (H) IHC staining for PSMD14 and IMPDH2 in brain tumor sections from the orthotopic xenografts. Scale bar = 100 μm. (I) Representative IF images of tumor tissues from the xenograft models. Scale bar = 100 μm. (J) Cell viability curves for GBM cells treated for 4 days with vehicle (DMSO), TMZ, thiolutin, or the combination of TMZ + thiolutin. (K) Quantification of cell viability (OD) at Day 4. Data are presented as mean ± SD (n = 3). ***P <0.001 (one-way ANOVA). (L) Bioluminescent imaging of intracranial tumors in mice treated with vehicle, TMZ, or TMZ + thiolutin on Days 7 and 28 post-implantation. (M) Quantification of photon flux at Day 28. Data are presented as mean ± SD (n = 5 per group). *P < 0.05, **P < 0.01, ***P < 0.001 (one-way ANOVA). (N) Representative IHC images of PSMD14 and IMPDH2 in tumor tissues from mice treated with TMZ alone or TMZ + thiolutin. Scale bar = 100 μm.

To investigate the therapeutic relevance of targeting the PSMD14-IMPDH2 axis, we first evaluated the anti-tumor activity of Thiolutin in GBM models. Thiolutin exhibited potent, dose-dependent suppression of cell viability in LN229, U118MG, and GBM#P3 cells, with IC50 values in the low micromolar range (Figure 6A). This cytotoxic effect was further supported by consistent IC50 measurements in dose-response curves (Figure 6B, C). Correspondingly, Thiolutin treatment resulted in dose-dependent reduction of IMPDH2 protein level (Figure S7A).

In orthotopic GBM models, Thiolutin significantly inhibited intracranial tumor growth, as shown by bioluminescence imaging and tumor flux quantification (Figure 6D, E), and extended overall survival compared to controls (Figure 6F). Histological assessment confirmed a marked reduction in tumor mass following Thiolutin administration (Figure 6G). Immunohistochemistry and immunofluorescence further demonstrated decreased expression of IMPDH2 in Thiolutin-treated tumors (Figure 6H, I). *In vitro*, Thiolutin suppressed DNA synthesis and induced apoptosis (Figure 6J, K**;** Figure S7B, C) 32, which could be partially reversed by overexpression of PSMD14, confirming on-target specificity (Figure S7D, E).

Cell-cycle analysis showed that Thiolutin induced robust G0/G1 phase arrest, which was similarly rescued by PSMD14 overexpression (Figure S8A, B). Consistent with *in vitro* findings, *in vivo* xenograft models treated with Thiolutin and PSMD14 overexpression exhibited significant differences in tumor growth kinetics, as visualized by longitudinal bioluminescence imaging (Figure S8C, D). Importantly, Thiolutin showed minimal off-target toxicity, with no evident histopathological damage in heart, lung, liver, kidney, or spleen (Figure S8E). We next evaluated the potential synergy between Thiolutin and the standard chemotherapeutic agent TMZ 17. Combined Thiolutin and TMZ treatment markedly enhanced tumor suppression *in vivo*, resulting in significantly decreased tumor radiance signals (Figure 6L, M) and lower expression levels of IMPDH2, as shown by IHC (Figure 6N). Mechanistically, the combination treatment amplified cell-cycle arrest, apoptosis, and necrosis compared to either agent alone (Figure S9A-D). In orthotopic GBM models, co-administration of Thiolutin and TMZ resulted in striking reductions in tumor growth and extended overall survival (Figure S9E-I). Together, these results underscore that dual inhibition of the PSMD14-IMPDH2 axis not only disrupts metabolic support for DNA repair but also sensitizes GBM cells to alkylating damage by TMZ. This strategy achieves a double-hit on GBM vulnerabilities-compromising purine biosynthesis and mitochondrial integrity while simultaneously amplifying genotoxic stress-providing a robust mechanistic rationale for combination-based therapeutic interventions 33.

## Discussion

Our study unravels an unprecedented coupling mechanism between protein post-translational modifications and metabolic homeostasis in GBM, spotlighting the crucial role of PSMD14. Historically the UPS has been primarily studied for its function in protein degradation 34; however, our findings significantly expand this perspective by revealing that PSMD14 directly stabilizes the rate-limiting enzyme for *de novo* guanine nucleotide biosynthesis 35, IMPDH2 through selective removal of K48-linked polyubiquitin chains 36. This regulatory axis is vital for sustaining nucleotide biosynthesis and mitochondrial energy metabolism, which are indispensable for rapid tumor cell proliferation and survival 37. Moreover, it highlights a novel intersection between PTM and metabolic pathways, offering a fresh angle to comprehend the intricate molecular underpinnings of GBM 4.

PSMD14 emerges as a critical, dual-functional target in GBM, wielding the capacity to modulate metabolic processes and oncogenic signaling. By stabilizing IMPDH2, PSMD14 ensures a continuous supply of nucleotides and robust mitochondrial function, thereby directly fueling GBM cell proliferation, survival, and invasive capability. In parallel, PSMD14 stabilizes multiple oncogenic proteins such as β-catenin, further driving malignant phenotypes and resistance to conventional therapies 38. Thus, targeting PSMD14 presents a uniquely advantageous therapeutic approach, capable of disrupting multiple pro-tumorigenic pathways simultaneously 39. Furthermore, our results validate PSMD14 as a potent therapeutic sensitizer, significantly heightening GBM cells' vulnerability to TMZ 40, the frontline chemotherapeutic agent. Given TMZ's limited efficacy due to inherent or acquired resistance, PSMD14 inhibition emerges as a promising adjunct strategy for overcoming therapeutic barriers and improving clinical outcomes.

Central to PSMD14's oncogenic impact is its downstream regulation of IMPDH2, whose critical function in nucleotide metabolism directly impacts tumor cell energetics and proliferation. Through precise deubiquitination, PSMD14 stabilizes IMPDH2, maintaining optimal GTP pools essential for nucleolar integrity, mitochondrial respiration, and cellular proliferation 41. Consistent with previous literature, our data demonstrate that impairing the PSMD14-IMPDH2 axis profoundly disrupts GBM cell metabolism, reducing oxidative phosphorylation and glycolysis 42, leading to ATP depletion, mitochondrial membrane potential collapse, and subsequent activation of DNA damage responses and apoptosis pathways. These metabolic vulnerabilities underscore the therapeutic potential of targeting nucleotide biosynthesis and mitochondrial function via PSMD14 inhibition 43.

The dual-target profile of Thiolutin, which primarily targets PSMD14 but is also reported to inhibit HDAC activity, presents both a unique therapeutic opportunity and potential complexity. Our findings predominantly implicate PSMD14 inhibition in Thiolutin's anti-GBM effects. Nevertheless, concurrent HDAC inhibition may confer additional anti-tumor activities, possibly enhancing efficacy or altering tumor susceptibility to chemotherapy. The precise contributions of these dual activities require further clarification. Future research should focus on dissecting these dual mechanisms, evaluating the pharmacokinetic properties, and improving the brain penetration of Thiolutin or developing more selective PSMD14 inhibitors with enhanced efficacy and minimized neurotoxicity 44.

Our study boasts several strengths. These include the comprehensive identification of PSMD14 as a key regulator in GBM through integrative transcriptomic and proteomic analyses, the elucidation of its novel regulatory mechanism on IMPDH2 stability, and the demonstration of its therapeutic potential in preclinical models. However, we acknowledge that our study also has limitations. These particularly pertain to the detailed molecular mechanisms governing substrate specificity of PSMD14 and whether additional substrates exist that significantly contribute to the observed phenotypes. Additionally, as a component of the proteasome complex, global PSMD14 inhibition might adversely impact normal cellular protein turnover. This necessitates comprehensive toxicological evaluations in further studies.

In conclusion, our study significantly propels the understanding of cancer metabolic regulation by identifying PSMD14 as a central nexus that integrates protein post-translational modifications with nucleotide metabolism and mitochondrial energy homeostasis. Targeting the PSMD14-IMPDH2 metabolic axis represents an innovative therapeutic approach, capable of disrupting tumor metabolism and sensitizing cancer cells to conventional chemotherapy. Our findings provide a robust experimental foundation for further clinical translation, highlighting PSMD14 inhibition, particularly via dual-target inhibitors such as Thiolutin, as a highly promising strategy in the targeted therapy against GBM.

## Supplementary Material

Supplementary figures and table.

## Abbreviations

GBM: Glioblastoma multiforme
DUBs: Deubiquitinating enzymes
IP-MS: immunoprecipitation coupled mass spectrometry
TMZ: temozolomide
UPS: ubiquitin-proteasome system
GTP: guanine triphosphate
Co-IP/MS: co-immunoprecipitation followed by mass spectrometry
DEGs: differentially expressed genes
OCR: Oxygen consumption rate
ECAR: extracellular acidification rate
