# Supplementary material for: Coupling proteostasis and de novo purine biosynthesis of PSMD14 fuels glioblastoma progression and chemoresistance
Source: Theranostics. 2026 Jan 1;16(7):3599–615. doi: 10.7150/thno.124409 (PMC12846754; doi:10.7150/thno.124409)
Supplement: Supplementary file 1 — Supplementary figures and table. [file thnov16p3599s1.pdf]

Supplementary Material

Coupling proteostasis and de novo purine biosynthesis of PSMD14 fuels glioblastoma progression and chemoresistance

Jiazheng Wang[1,2†], Qun Cao[1,2†], Zhikai Li[3], Xuxiu Lu[1,2,4], Zhuo Li[1,2], Chenghui Guo[1,2], Yuan Pan[5], Qing Zhang[1,2], Wenjie Li[1,2], Guo Xiang[1,2], Anjing Chen[1,2*]

[1] Department of Neurosurgery, Qilu Hospital, Cheeloo College of Medicine and Institute of Brain and Brain-Inspired Science, Shandong University, Jinan 250012, Shandong, China

[2] Jinan Microecological Biomedicine Shandong Laboratory and Shandong Key Laboratory of Brain Health and Function Remodeling, Jinan 250012, Shandong, China

[3] Department of Radiation Oncology, Cheeloo College of Medicine
Shandong University, Jinan 250012, Shandong, China

[4] Rizhao People's Hospital, Rizhao 276800, Shandong Province, China

[5] Medical Integration and Practice Center, Cheeloo College of Medicine, Shandong University, Jinan, China.

† These authors contribute equally to this work.

* Corresponding Author: Anjing Chen, Ph.D.

Department of Neurosurgery, Qilu Hospital, Cheeloo College of Medicine and Institute of Brain and Brain-Inspired Science, Shandong University, Jinan, 250012, China

E-mail: chenaj@sdu.edu.cn

Table S1

Proteins interacting with PSMD14 detected by MS.

MYH9	TGM2	RPL6	RPL7A	RPS2
MYH10	ALB	RPS8	CALML5	HSPA9
ACTG1	S100A8	LACTB	ATP5F1B	RPL15
VIM	MYO18A	TOP2A	EEF1A2	HMGA2
FN1	GRN	IGKV2-40	RPL39	HSP90AB1
ACTA1	TPM4	TRAP1	ANKHD1	RPL36AL
TUBB	S100A7	GAPDH	XRCC5	CSE1L
MYO1C	S100A9	XRCC6	ENO1	RPL36AL
TUBA1B	COL1A2	RPS29	HSP90AA1	Actin, cytoplasmic 1
IMPDH2	RPL3	ACTN1	PLEC	HSP 90-alpha
MYO5A	RPL4	Immunoglobulin	RPLP1	HSP 75 kDa, mitochondrial
MVP	RPL13	LMNA	RPL8	Clathrin heavy chain 1
MYL6	MYO1B	HMGA1	RPL14	Progranulin
HRNR	ATP5F1A	MYO6	TXN	Myosin regulatory light chain 12B
MYL12B	TPM3	DBT	THOC7	Unconventiona l myosin-VI

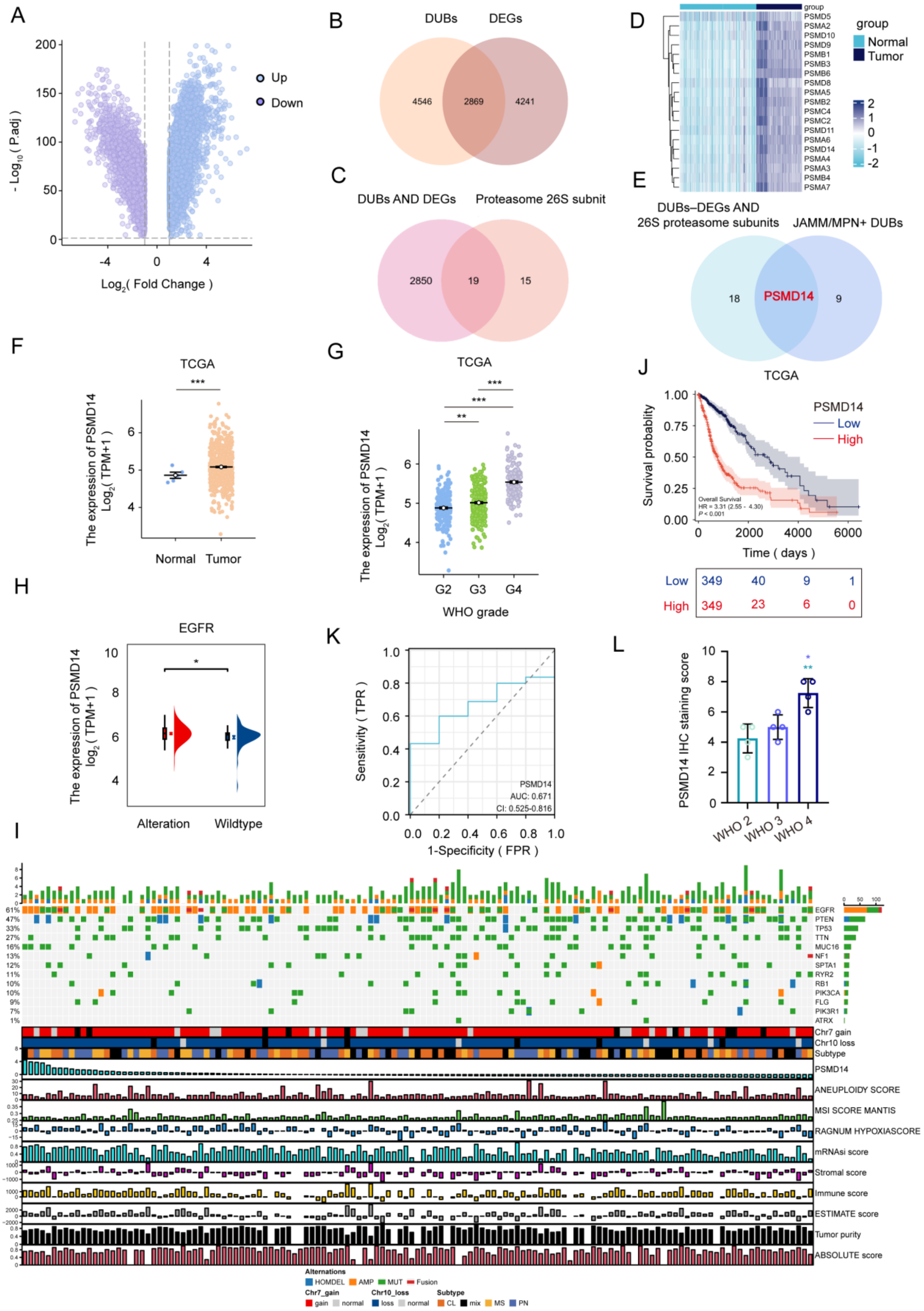

Figure S1

Elevated expression of PSMD14 is associated with glioblastoma malignancy and poor patient prognosis. (A) Volcano plot of RNA-seq–derived differential gene expression between normal brain tissue and GBM (TCGA). (B) Venn diagram showing

overlap between genes encoding deubiquitinating enzymes (DUBs) and GBM differentially expressed genes (DEGs). (C) Venn diagram of DUB genes and 26S proteasome subunit genes found among the DEGs. (D) Heatmap of log_2-transformed expression levels of 26S proteasome subunit genes in normal versus GBM samples (TCGA). Expression values were z-score-normalized across samples. (E) Venn diagram highlighting PSMD14 as the sole DUBs gene at the intersection of the DEG set and the 26S proteasome components. (F) Box-and-whisker plot of PSMD14 mRNA expression (log_2TPM) in normal brain versus GBM tissues (TCGA); Data are presented as mean ± SD. ***P < 0.001 (Independent-sample Student-T test). (G) Scatter plot of PSMD14 expression across WHO glioma grades II, III, and IV; Data are presented as mean ± SD. ***P < 0.001 (one-way ANOVA). (H) Bar graph of PSMD14 expression (log_2TPM) in GBM molecular subtypes. Data are presented as mean ± SD. *P < 0.05 (one-way ANOVA). (I) Complex heatmap of GBM sample mutation status, chromosome structure, subtype, expression of PSMD14, and GSVA scores. Data were obtained from TCGA. (J) Kaplan–Meier survival curves for GBM patients stratified by low versus high PSMD14 expression (TCGA). (K) Receiver-operator characteristic (ROC) curve for PSMD14 as a classifier of GBM versus normal brain. (L) Quantification of PSMD14 IHC signal (H-score) in xenograft tumor tissues across multiple fields (n = 4 per group). Data are presented as mean ± SD. *P < 0.05, **P < 0.01 (one-way ANOVA).

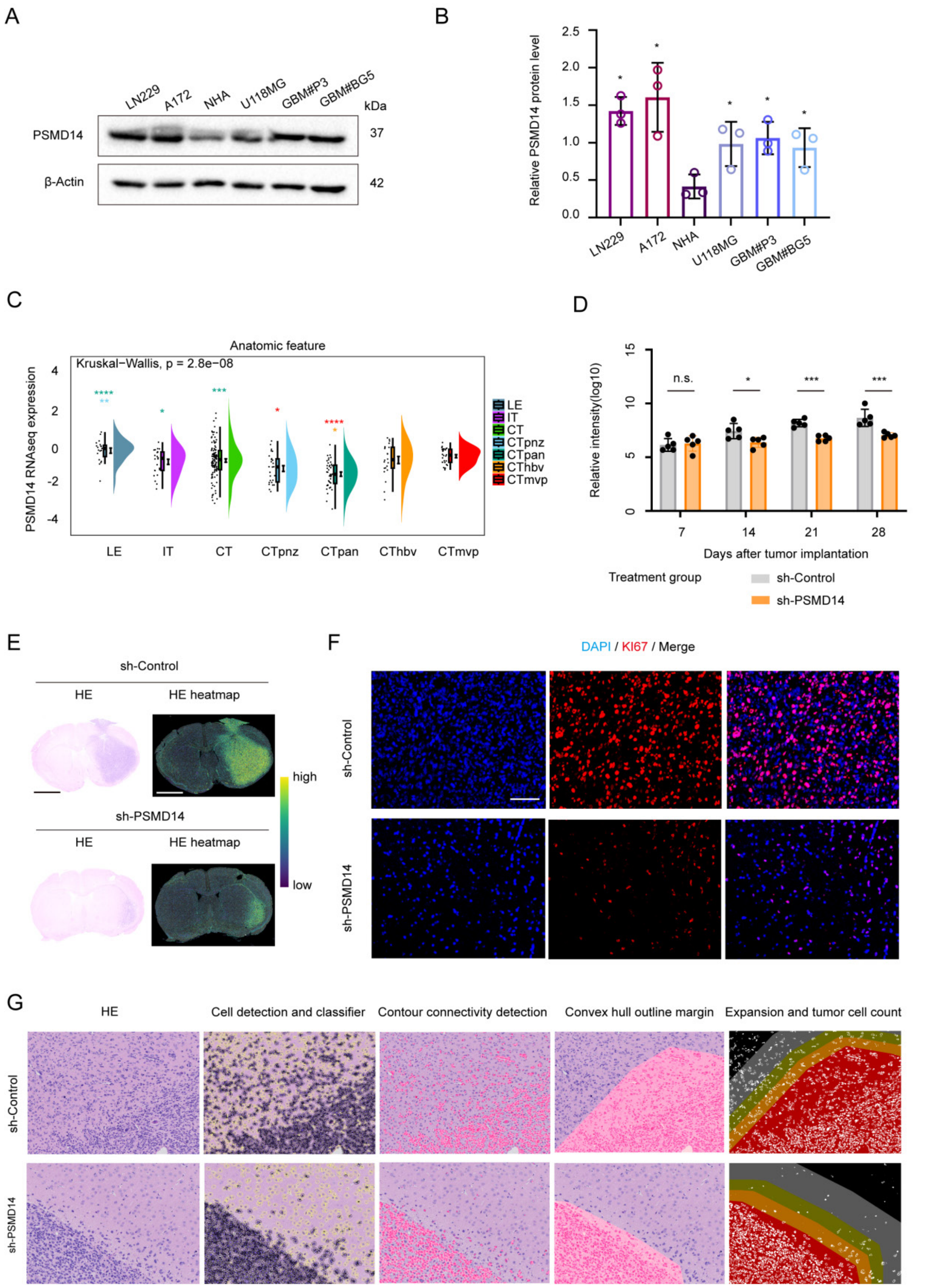

Figure S2

PSMD14 is highly expressed in GBM cells and tumor niches and promotes intracranial tumor growth in vivo. (A) Western blot of PSMD14 protein in GBM cell lines. (B) Statistical analysis of normalized PSMD14 protein levels based on the Western blot results. Data are presented as mean ± SD (n = 3). *P < 0.05 (one-way ANOVA). (C)

Violin plots showing PSMD14 RNA-seq expression across distinct anatomic transcriptional features of GBM (TCGA). Significant differences were observed among leading edge (LE), infiltrating tumor (IT), cellular tumor (CT), and microvascular proliferation regions; CTpan denotes pseudopalisading cells around necrosis, CTpnz denotes the perinecrotic zone, CTbv denotes cellular tumor with hyperplastic/blood-vessel–rich regions, and CTmvp denotes cellular tumor with microvascular proliferation. (D) Quantification of photon flux ($log_{10}p/s/cm^2/sr$) (mean ± SD, n = 5 per group); Data are presented as mean ± SD. *P < 0.05, ***P < 0.001 (Independent-sample Student-T test). (E) Representative HE-stained brain sections and HE heatmap from sh-Control and sh-PSMD14 xenograft tumors. Scale bar = 2.5 mm. (F) Representative immunofluorescence (IF) images of GBM xenograft tumor sections labeled for Ki-67 in sh-Control versus sh-PSMD14 groups. Scale bar = 100 μm. (G) Schematic of the image analysis pipeline used for tumor histology.

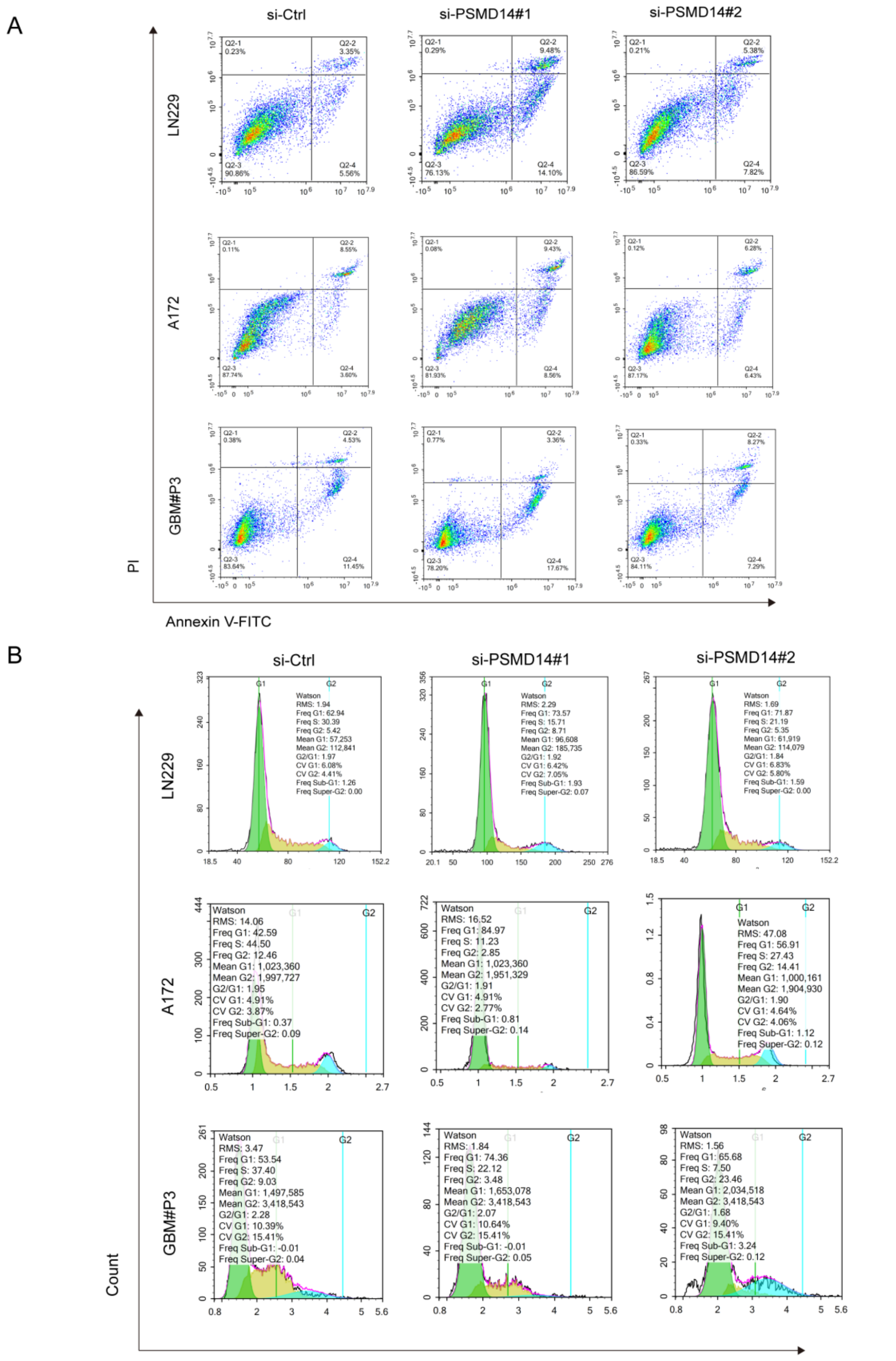

Figure S3

Knockdown of PSMD14 induces apoptosis and cell cycle arrest in GBM cells. (A) Representative flow cytometry dot plots (Annexin V–FITC/PI) showing apoptosis analysis

in LN229, A172, and GBM#P3 cells. (B) Representative cell cycle histograms for LN229, A172, and GBM#P3 cells.

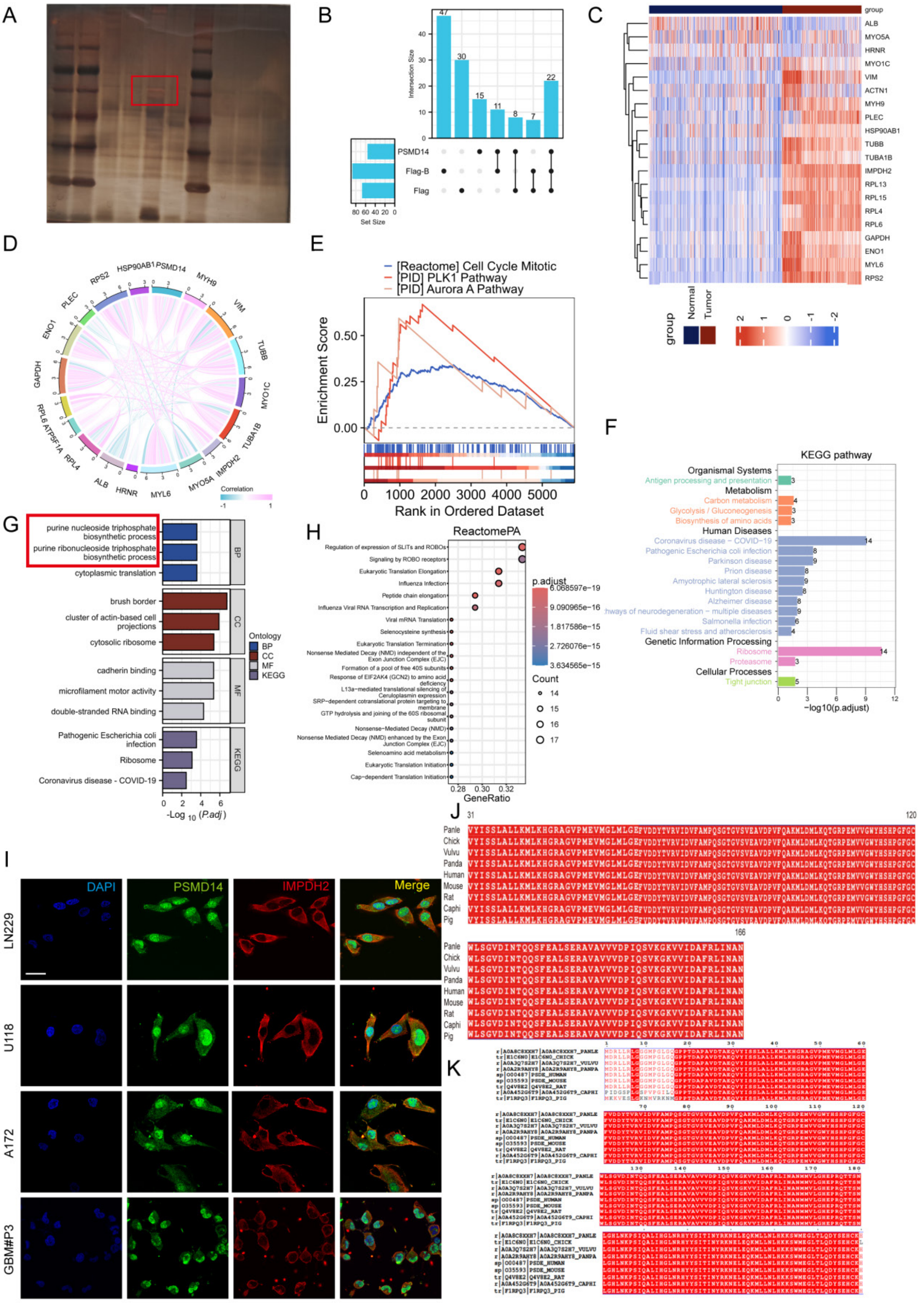

Figure S4

Integrative interactome analyses identify IMPDH2 as a conserved PSMD14 interactor and reveal associated metabolic networks in GBM. (A) Protein silver stain of PSMD14 and IMPDH2. (B) UpSet plot summarizing the number of unique and shared proteins

identified by MS in PSMD14, Flag-B, and Flag datasets. (C) Heatmap of log_2-normalized RNA-seq expression (TPM) for candidate PSMD14-interacting proteins in normal brain versus GBM tissues (TCGA). Values are z-score–normalized. (D) Circos diagram of the PSMD14 co-expression network. (E) Gene set enrichment analysis (GSEA) plots for PSMD14-correlated genes. (F) KEGG pathway enrichment analysis for PSMD14-correlated genes. (G) Enriched Gene Ontology (GO) terms for PSMD14-correlated genes, with separate lists for Biological Process (BP), Cellular Component (CC), Molecular Function (MF), and KEGG. (H) Dot plot of Reactome pathway enrichment for PSMD14-correlated genes. (I) Confocal images of GBM cell lines (LN229, U118, A172, GBM#P3) stained for PSMD14 and IMPDH2. Scale bar = 30 μm. (J) Multiple sequence alignment of PSMD14 protein across species. (K) Multiple sequence alignment of IMPDH2 protein across species.

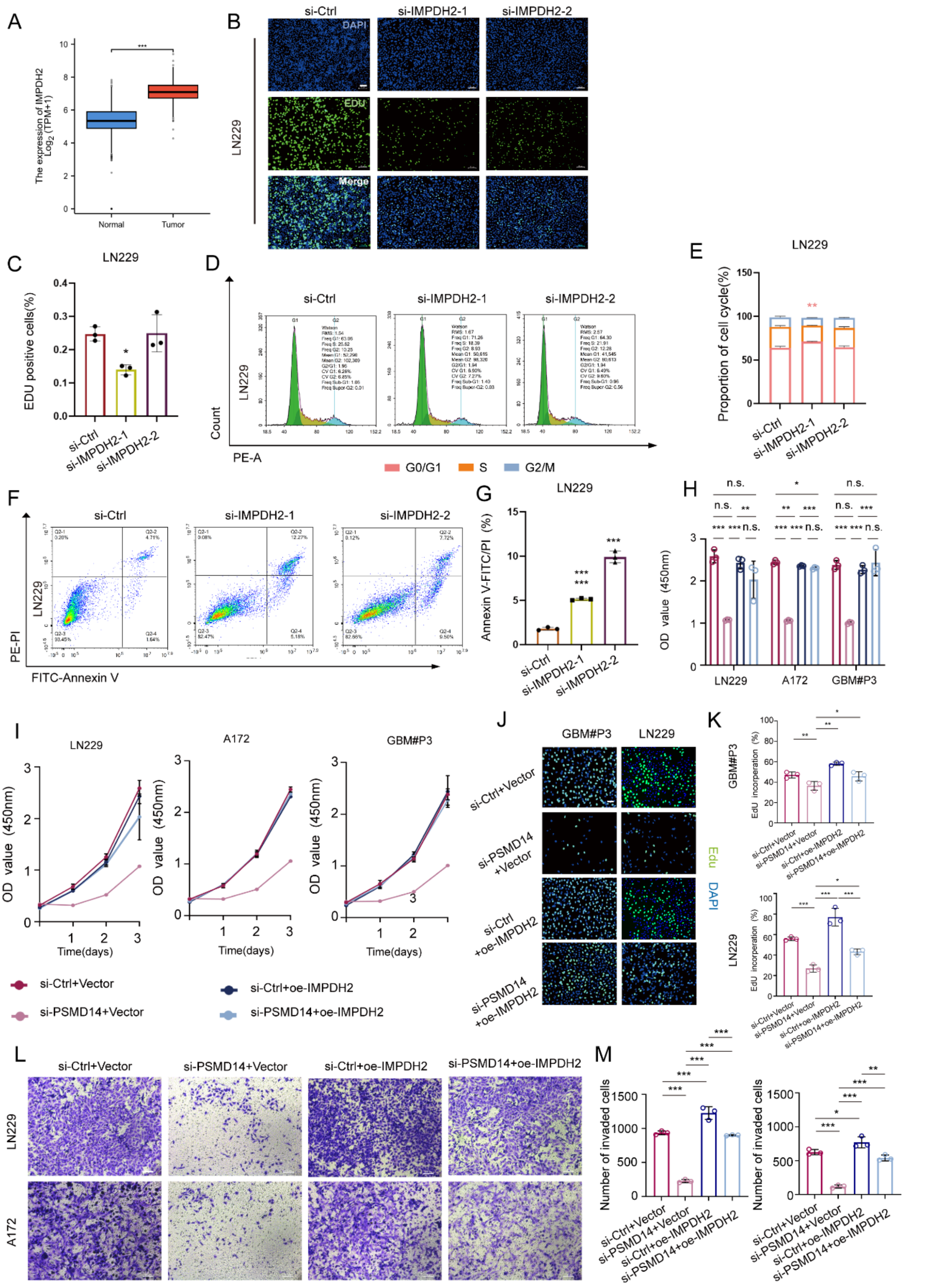

Figure S5

IMPDH2 is upregulated in GBM and functionally cooperates with PSMD14 to promote proliferation, survival, and invasion. (A) Box-and-whisker plot of IMPDH2 mRNA expression (log_2TPM) in normal brain versus GBM samples (TCGA). Data are presented

as mean ± SD. ***P < 0.001 (Independent-sample Student-T test). (B) Representative images of EdU incorporation in LN229 cells transfected with control siRNA or IMPDH2-targeting siRNAs. Scale bar =100 μm. (C) Quantification of EdU positive cells. Data are presented as mean ± SD (n = 3). *P < 0.05 (one-way ANOVA). (D) Representative cell cycle histograms for LN229 cells, indicating cell cycle arrest (G0/G1, S, G2/M). (E) Quantification of cell cycle phase distribution for LN229. The statistical comparisons and asterisks specifically refer to the G0/G1 fraction among the three groups. Data are presented as mean ± SD (n = 3). **P < 0.01 (one-way ANOVA). (F) Representative flow cytometry dot plots (Annexin V–FITC/PI) for LN229 cells with control or IMPDH2 siRNAs. (G) Quantification of apoptotic cells. Data are presented as mean ± SD (n = 3). ***P < 0.001 (one-way ANOVA). (H) CCK-8 cell viability measured on Day 3 for LN229, A172, and GBM#P3. Data are presented as mean ± SD (n = 3). *P < 0.05, **P < 0.01, ***P < 0.001 (one-way ANOVA). (I) Time-course of cell viability for LN229, A172, GBM#P3 transfected with: si-Ctrl+Vector, si-PSMD14+Vector, si-Ctrl+oe-IMPDH2, si-PSMD14+oe-IMPDH2. Each growth curve is mean ± SD (n = 3). (J) Representative images of EdU in GBM#P3 and LN229 cells. Scale bar = 100 μm. (K) Quantification of EdU positive cells for GBM#P3 and LN229. Data are presented as mean ± SD (n = 3). *P < 0.05, **P < 0.01, ***P < 0.001 (one-way ANOVA). (L) Representative Transwell invasion assay images for LN229 and A172. Scale bar = 100 μm. (M) Quantification of invaded cells. Data are presented as mean ± SD (n = 3). *P < 0.05, **P < 0.01, ***P < 0.001 (one-way ANOVA).

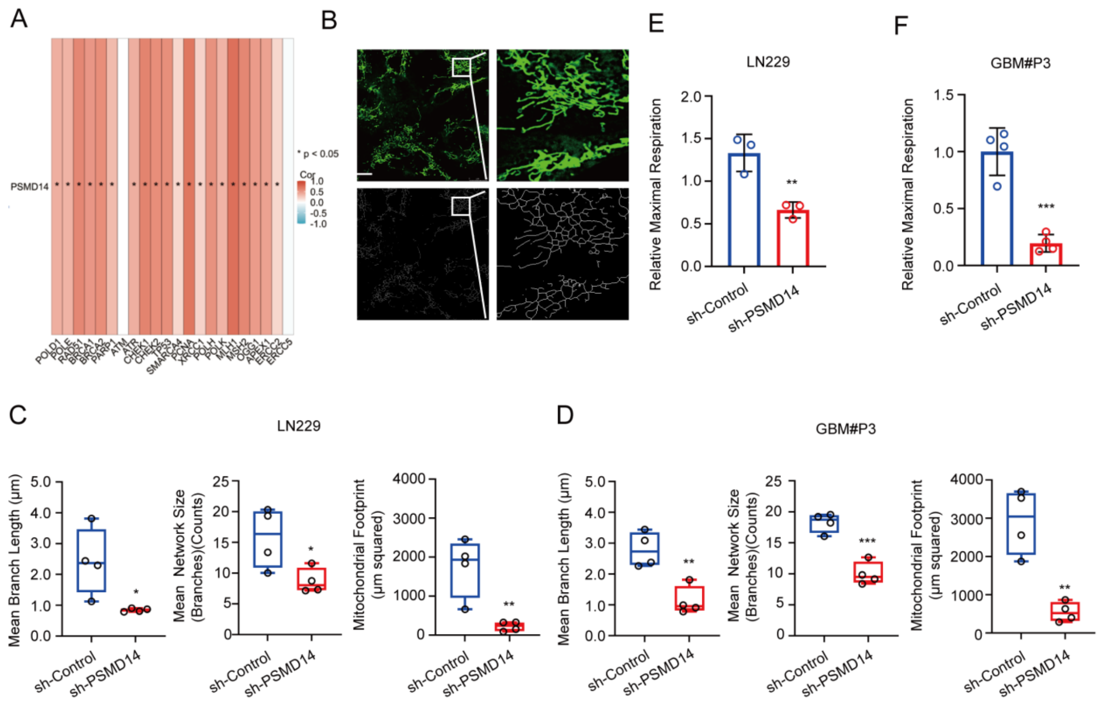

Figure S6

PSMD14 knockdown correlates with DNA damage signaling and disrupts mitochondrial morphology and respiration in GBM cells. Heatmap of correlation (Pearson's r) between PSMD14 expression and key DNA damage genes in GBM. Asterisk indicates correlations reaching P< 0.05. (B) Confocal microscopy of LN229 cells stained with MitoTracker Green. Scale bar = 30 μm. (C-D) Mitochondrial network analysis in LN229 and GBM#P3 cells. Data are presented as mean ± SD (n = 4). *P < 0.05，**P < 0.01, ***P < 0.001 (Independent-sample Student-T test). (E-F) Seahorse extracellular flux analysis: bar graph of relative maximal respiration (OCR) in LN229 and GBM#P3 cells. Data are presented as mean ± SD (n = 3). **P < 0.01, ***P < 0.001 (Independent-sample Student-T test).

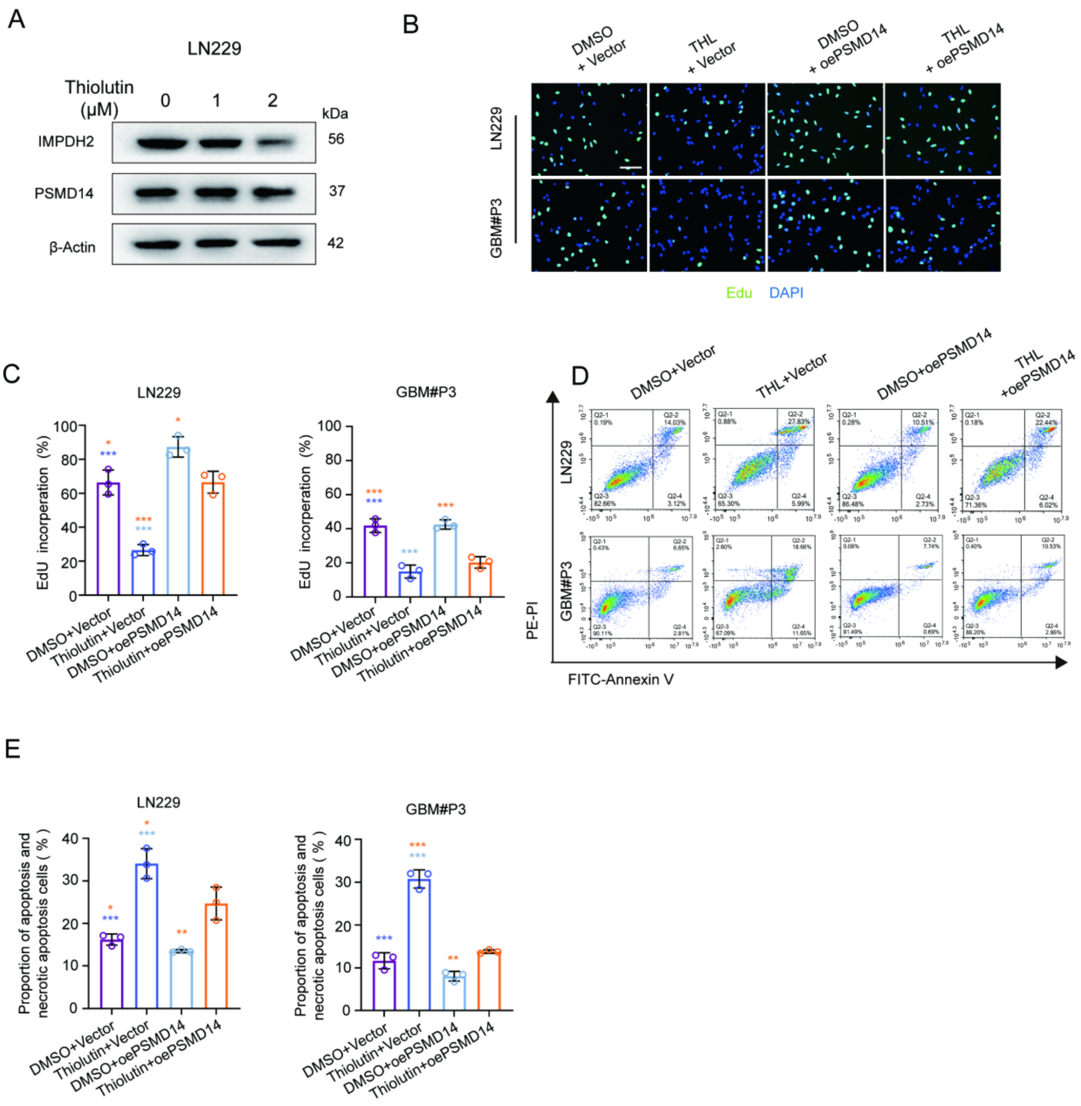

Figure S7

Thiolutin suppresses GBM cell proliferation and induces apoptosis through PSMD14 inhibition, effects partially rescued by PSMD14 overexpression. (A) Western blot of LN229 cells treated with Thiolutin. (B) Representative images of EdU in LN229 and GBM#P3 cells treated with DMSO or Thiolutin, with or without PSMD14 overexpression. Scale bar = 100 μm. (C) Quantification of EdU positive cells for LN229 and GBM#P3. Data are presented as mean ± SD (n = 3). ***$P < 0.001$ (one-way ANOVA). (D) Representative flow cytometry dot plots (Annexin V–FITC/PI) for LN229 and GBM#P3. (E) Quantification of apoptotic cells. Data are presented as mean ± SD (n = 3). *$P < 0.05$, **$P < 0.01$, ***$P < 0.001$ (one-way ANOVA).

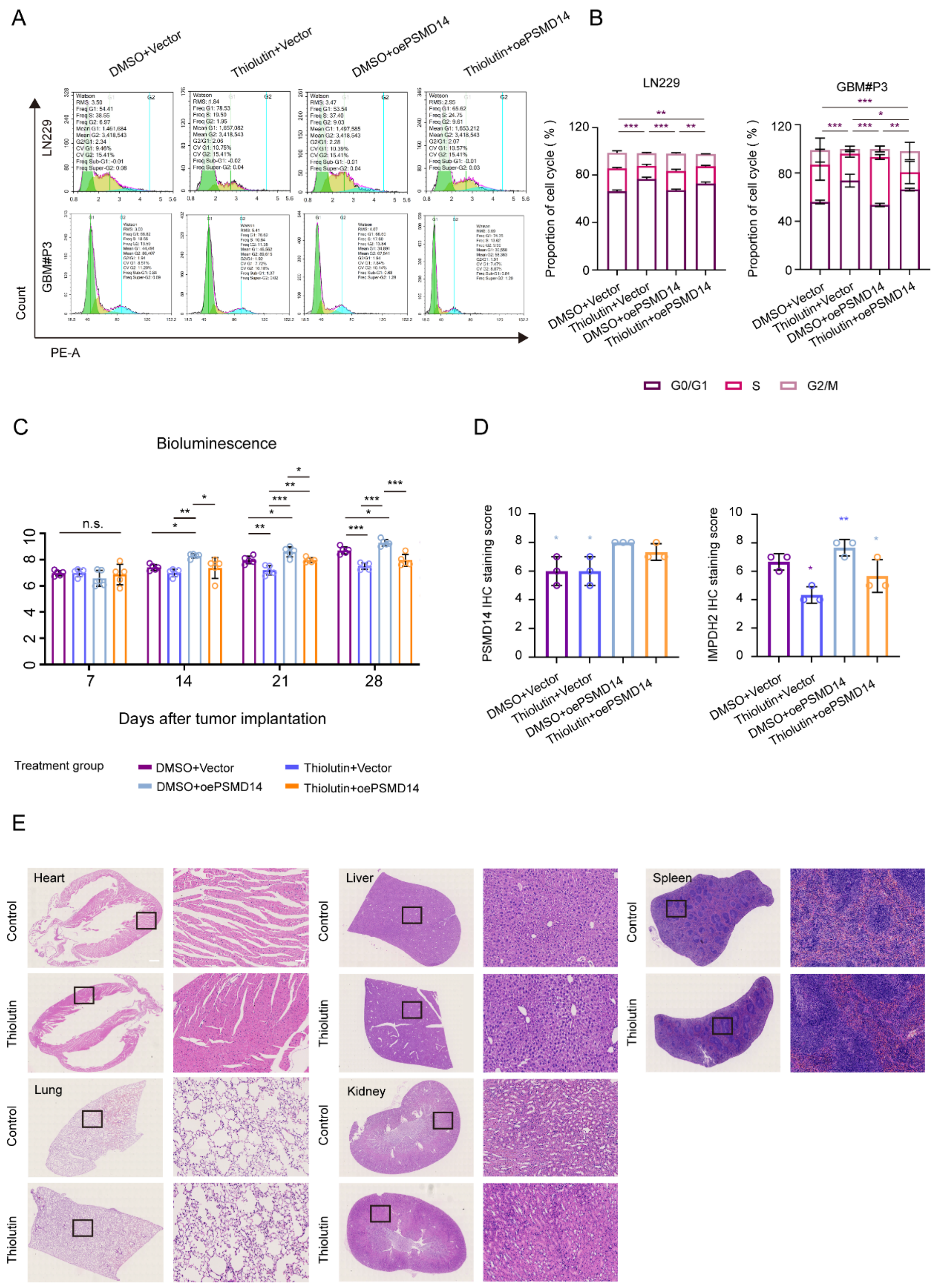

Figure S8

Thiolutin induces G0/G1 arrest and suppresses GBM growth with minimal systemic toxicity. (A) Representative cell cycle histograms for LN229 and GBM#P3. (B) Quantification of cell cycle phase distribution LN229 and GBM#P3. The statistical

comparisons and asterisks specifically refer to the G0/G1 fraction among the four groups. Data are presented as mean ± SD (n = 3). *P < 0.05, **P < 0.01, ***P < 0.001 (one-way ANOVA). (C) Quantification of bioluminescence (log_{10} total photon flux) over time. Data are presented as mean ± SD (n = 5 per group). *P < 0.05, **P < 0.01, ***P < 0.001 (one-way ANOVA). (D) IHC H-scores for PSMD14 and IMPDH2 in tumor sections, across treatment groups. Data are presented as mean ± SD (n = 3 per group). *P < 0.05, **P < 0.01 (one-way ANOVA). (E) Histological examination of major organs from treated mice: representative HE images of heart, lung, liver, kidney, and spleen from control versus thiolutin-treated mice. Scale bars = 100 μm.

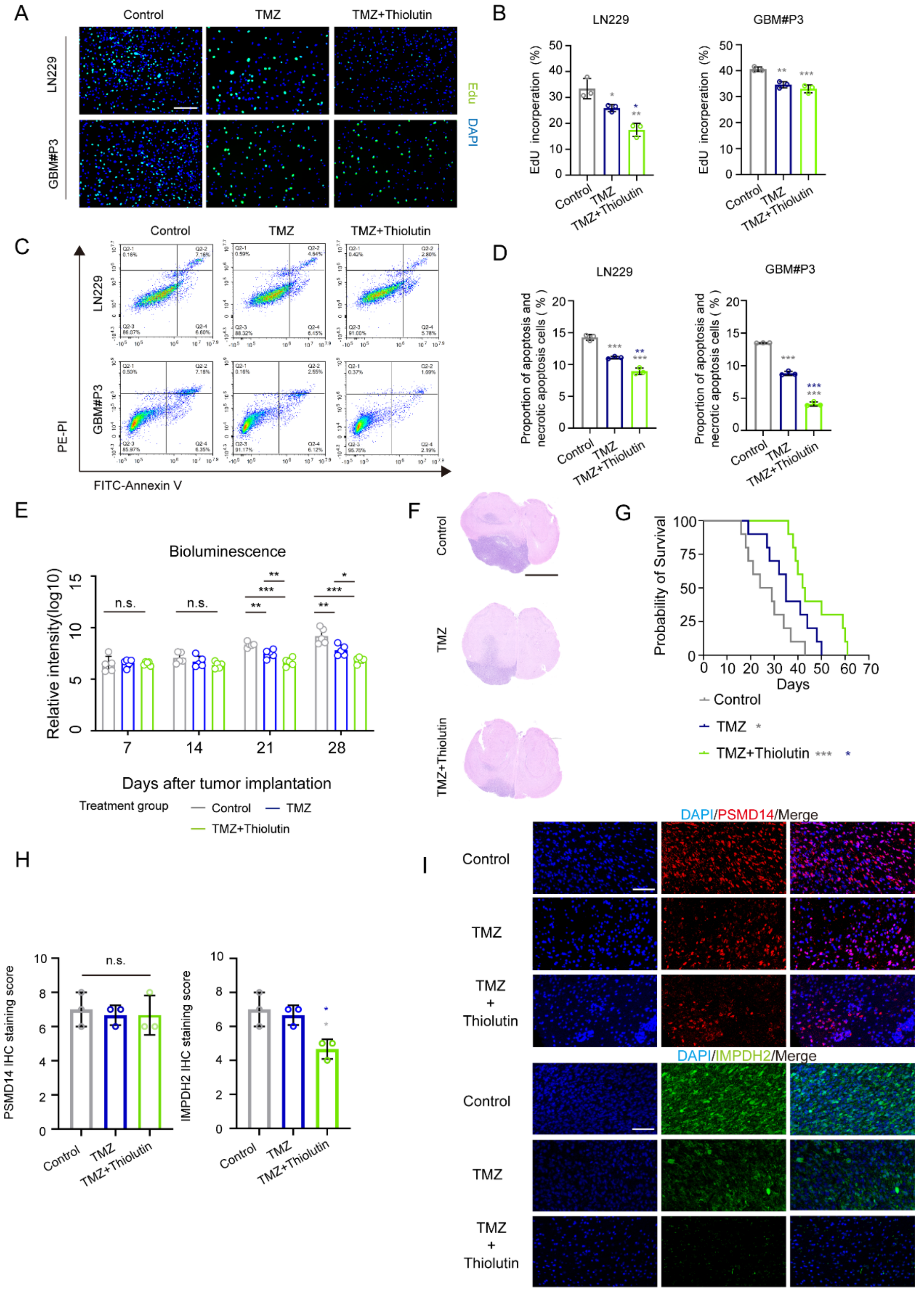

Figure S9

Thiolutin synergizes with temozolomide to suppress GBM growth, induce apoptosis, and prolong survival. (A) Representative images of EdU incorporation assays in LN229 and GBM#P3 cells treated with vehicle control, TMZ, or TMZ in combination with Thiolutin.

Scale bar = 100 μm. (B) Quantification of EdU-positive cells as a percentage of total nuclei. Data are presented as mean ± SD (n = 3). *P < 0.05, **P < 0.01, ***P < 0.001 (one-way ANOVA). (C) Representative flow cytometry dot plots (Annexin V–FITC/PI) in LN229 and GBM#P3 cells. (D) Quantification of apoptotic cells. Data are presented as mean ± SD (n = 3). **P < 0.01, ***P < 0.001 (one-way ANOVA). (E) In vivo bioluminescence imaging analysis of orthotopic xenografts at days 7, 14, 21, and 28 post-implantation. Total luminescence intensity was quantified. Data are presented as mean ± SD (n = 5 per group). *P < 0.05, **P < 0.01, ***P < 0.001 (one-way ANOVA). (F) Representative hematoxylin and eosin (HE) staining of coronal brain sections from orthotopic xenograft mice. Scale bar = 2.5 mm. (G) Kaplan–Meier survival curves of mice bearing intracranial GBM xenografts. Data are presented as mean ± SD (n = 10 per group). *P < 0.05, ***P < 0.001 (one-way ANOVA). (H) Quantitative analysis of immunohistochemical (IHC) staining scores for PSMD14 and IMPDH2 in brain tumor tissues. Data are presented as mean ± SD (n = 3). *P < 0.05, ***P < 0.001 (one-way ANOVA). (I) Representative immunofluorescence (IF) staining of PSMD14 or IMPDH2 in tumor sections from each treatment group. Scale bar = 100 μm.
